# Dissecting the Subcellular Localization, Intracellular Trafficking, Interactions, Membrane Association, and Topology of Citrus Leprosis Virus C Proteins

**DOI:** 10.3389/fpls.2018.01299

**Published:** 2018-09-11

**Authors:** Mikhail Oliveira Leastro, Elliot Watanabe Kitajima, Marilia Santos Silva, Renato Oliveira Resende, Juliana Freitas-Astúa

**Affiliations:** ^1^Departamento de Bioquímica Fitopatológica, Instituto Biológico, São Paulo, Brazil; ^2^Departamento de Fitopatologia e Nematologia, Escola Superior de Agricultura Luiz de Queiroz, Universidade de São Paulo, Piracicaba, Brazil; ^3^Laboratório de Bioimagem, Embrapa Recursos Genéticos e Biotecnologia, Brasilia, Brazil; ^4^Departamento de Biologia Celular, Universidade de Brasilia, Brasilia, Brazil; ^5^Embrapa Mandioca e Fruticultura, Cruz das Almas, Bahia, Brazil

**Keywords:** cilevirus, CiLV-C, confocal microscopy, viral protein sub-localization, protein trafficking, virus assembly and replication, viral protein interaction, membrane protein topology

## Abstract

Citrus leprosis (CL) is a re-emergent viral disease affecting citrus crops in the Americas, and citrus leprosis virus C (CiLV-C), belonging to the genus *Cilevirus*, is the main pathogen responsible for the disease. Despite the economic importance of CL to the citrus industry, very little is known about the performance of viral proteins. Here, we present a robust *in vivo* study around functionality of p29, p15, p61, MP, and p24 CiLV-C proteins in the host cells. The intracellular sub-localization of all those viral proteins in plant cells are shown, and their co-localization with the endoplasmic reticulum (ER), Golgi complex (GC) (p15, MP, p61 and p24), actin filaments (p29, p15 and p24), nucleus (p15), and plasmodesmata (MP) are described. Several features are disclosed, including i) ER remodeling and redistribution of GC apparatus, ii) trafficking of the p29 and MP along the ER network system, iii) self-interaction of the p29, p15, and p24 and hetero-association between p29-p15, p29-MP, p29-p24, and p15-MP proteins *in vivo*. We also showed that all proteins are associated with biological membranes; whilst p15 is peripherally associated, p29, p24, and MP are integrally bound to cell membranes. Furthermore, while p24 exposes an N-cytoplasm-C-lumen topology, p29, and p15 are oriented toward the cytoplasmic face of the biological membrane. Based on our findings, we discuss the possible performance of each protein in the context of infection and a hypothetical model encompassing the virus spread and sites for replication and particle assembly is suggested.

## Introduction

Citrus leprosis is considered one of the main viral diseases affecting citrus orchards distributed from South to North America. It was first reported at the beginning of the 20th century causing considerable economic losses in US and, soon after, in the South of South America (Childers et al., 2003a; Bastianel et al., 2010; Ramos-González et al., 2016). This re-emergent disease is caused by leprosis-associated viruses from three genera (*Cilevirus, Dichorhavirus*, and *Higrevirus*). Currently, citrus leprosis virus C (CiLV-C) is considered the most devastating virus infecting citrus in Brazil (Bastianel et al., 2010). Although described more than a century ago, the viral nature of citrus leprosis was only established around 1970's, being completed with the first sequencing of the CiLV-C genome in 2006 (Knorr, 1968; Kitajima et al., 1974; Locali-Fabris et al., 2006; Pascon et al., 2006; Bastianel et al., 2010). Since then, the virus was described in more than 50 natural and experimental host species belonging to at least 28 plant families (Garita et al., 2014; Arena et al., 2016). The wide distribution of the disease is due to the worldwide expansion of its vector, *Brevipalpus yothersi* (Acari: *Tenuipalpidea*) (Childers et al., 2003b; Beard et al., 2015). The interaction between CiLV-C and *B. yothersi* occurs in a persistent manner; however, while some authors suggest that the virus propagates in the mite, based on the finding of the supposedly complementary viral RNA in the vector (Roy et al., 2015), others propose it only circulates in the vector, because of the short latent period for transmission, the ability of larval forms to transmit the virus, and the absence of cytopathic effects suggestive of viral replication in mite tissues (Tassi et al., 2017). CiLV-C infection is characterized by the formation of chlorotic and necrotic circular lesions in citrus leaves, fruits, and stems (Garita et al., 2013, 2014; Ramos-González et al., 2016). A peculiarity of the infection is the limitation of systemic viral spread from the inoculation site by the vector to other regions of the plant, an intriguing phenotype rarely observed in most plant-virus interactions.

CiLV-C constitutes the type species of the genus *Cilevirus* (Locali-Fabris et al., 2012). Its genome is composed of two linear positive sense ssRNA segments with the presence of 5′ Cap and 3′ Poly(A) tail, organized in six open reading frames (ORF). The first segment (RNA1) has two ORF that encode an RNA-dependent RNA polymerase (RdRp), containing conserved domains of methyl transferase, helicase, protease and polymerase (Pascon et al., 2006), as well as a putative capsid protein, the p29. The second segment (RNA2) encodes: the p15 with unknown function and absence of similarity to any known gene; the p61, which exhibit features of a possible glycoprotein (Kuchibhatla et al., 2014); the putative movement protein (MP) that shares conserved motifs with the 30K MP superfamily (Pascon et al., 2006); and the p24 protein that shows a remote homology with a class of putative virion membrane protein of viruses from plants (blunervirus and higrevirus) and insects (negevirus) (Kuchibhatla et al., 2014).

In view of this diversity and mainly due to the restriction in their genome sizes, viral proteins typically play a multifunctional role in many aspects of the infection, which characterizes distinct functions for a single protein. Nonetheless, viral proteins also play common roles. In general, regardless of the virus class and host type, all viruses show common functional and structural properties. In most viruses, their genomes are encapsulated by a protective coat protein (Xue et al., 2014), which is also involved with genome assembly into ribonucleoprotein complex (vRNPs) for transcription and replication (Bol, 2008). Enveloped viruses contain membrane proteins associated to an additional lipid envelope of cellular origin; often glycoproteins that have been demonstrated to have important roles in virus transmission, relationship with vector species, infection, and immunity (Kikkert et al., 2001; Banerjee and Mukhopadhyay, 2016). In some cases, below the viral envelope is located the structural matrix protein, known to play a critical role in virion assembly and budding, interaction with RNPs as well as with the viral membrane; furthermore, it is responsible for the structural stability of virus particle (Neumann et al., 2009; Battisti et al., 2012). Additionally, viruses encode non-structural proteins, i.e., the gene silencing suppressor proteins that assist the success of viral infection acting against the mechanism of the host RNA silencing (Voinnet et al., 1999; Voinnet, 2002) and MPs, ensuring viral spread to neighbor cells and, sometimes, to systemic regions of the plant (Wolf et al., 1989; Lucas, 2006; Ueki, 2007).

In addition to these functions, viral proteins also act modifying the host machinery to ensure a successful infection. In this sense, most (+)RNA viruses use cell endomembranes as a way to escape of the action of plant defense machinery (Laliberté and Sanfaçon, 2010). Viral membrane-associated proteins may induce alteration of membrane morphology (Laliberté and Sanfaçon, 2010), and the formation of vesicle-like structures, which almost always correspond to sites for RNA replication and virion assembly (viral replication complex-VRCs) (Schwartz et al., 2004; Adams and Antoniw, 2005; Mandahar, 2006; Laliberté and Sanfaçon, 2010). In order to ensure viral spread, VRCs, structures as vRNPs may move intracellularly and to neighboring cells through plasmodesmata (PD) by the joint action of the viral proteins with microtubules and ER/actin network (Kawakami et al., 2004; Hofmann et al., 2009; Sambade and Heinlein, 2009; Niehl and Heinlein, 2011; Niehl et al., 2013).

The multifunctionality of viral proteins allows them to work in a synchronized manner, between them and in combination with host proteins, manipulating the cellular structure, thus ensuring the infection. In this sense, elucidating molecular aspects of virus proteins opens new scope for understanding the functional processes involved in the viral infection, an aspect that may contribute, in the long run, to the development of anti-viral treatments.

At present, little is known about the biological function of CiLV-C proteins. Given the importance of the disease, a detailed understanding of the molecular processes involved in the manifestation of citrus leprosis is still needed. In this context, the present work, for the first time, tries to decipher molecular details around the functionality of all (except for the polymerase) proteins coded by CiLV-C, based upon a robust *in vivo* study. We dissected the intracellular localization, property of intracellular trafficking, protein-protein interaction, membrane association, and topology of its proteins. Collectively, our findings allow us to infer the structural and non-structural roles of the proteins in the virion composition, and a broad discussion was generated from the possible performance of each protein in the context of infection, which allowed us to suggest a hypothetical model encompassing the virus spread and sites for replication and particle assembly.

## Materials and methods

### Plant maintenance and mite transmission

Five adult *Brevipalpus yothersi* mites from viruliferous colonies maintained on CiLV-C-infected sweet orange fruits were transferred to two or four leaves of *Nicotiana occidentalis, Abelmoschus esculentus, Portulaca oleracea*, and *Glycine max* plants. Before transferring the mites, the leaves of the assayed plants were carefully cleaned with cotton soaked in 70% ethanol, then washed with distilled water. Tanglefoot entomological glue was applied in the petiole to avoid the escape of the mites from the leaves where they were transferred to. At least three plants per species were assayed. The plants were kept under controlled conditions throughout the experiment. Plants were evaluated daily for the development of symptoms.

### Transmission electron microscopy (TEM)

For TEM, small fragments from the leaf lesions, including the tissue next to the lesions, were fixed in a mixture of 2.5% glutaraldehyde and 2% paraformaldehyde in 0.05M pH 7.2 cacodylate buffer (EMS) for at least 1 h, post-fixed in 1% OsO_4_ (EMS) (Kitajima and Nome, 1999), dehydrated in ethanol and embedded in Spurr's epoxy resin (EMS). Thin sections of embedded tissues were cut in a Leica UCT ultramicrotome with diamond knife, mounted on copper grids, stained with 3% uranyl acetate (EMS) and Reynold's lead citrate and examined under a Zeiss EM900 or JEOL JEM 1011 transmission electron microscopes. Leaf tissues from non-inoculated healthy plants were prepared similarly and examined as controls (data not shown).

### DNA manipulation and organelle markers

*p29, p15, p61, MP*, and *p24* genes of citrus leprosis virus C were amplified from total RNA extracted from infected citrus leaves and fruits (Sambrook and Russell, 2001). RT-PCR was processed following the manufacturer's specification (Invitrogen^TM^). The viral cDNA was generated with oligo (dT) primer that annealed to the viral extreme 3′ Poly(A). From cDNA, the gene amplification was performed with specific primers carrying the *Nco*I/*Nhe*I (*p61* and *MP*), *Pci*I/*Nhe*I (*p15* and *p24*), or *BspH*I/*Nhe*I (*p29*) sites.

To evaluate the sublocalization of the proteins, all the amplified genes were fused either at their C- or N-terminal with eGFP. For C-terminal fusion, the genes were cloned into the vector pSK35S-GFP:eGFP-PoPit (Herranz et al., 2005) replacing the GFP gene. The resultant clone pSK35S-p29:eGFP-PoPit, pSK35S-p15:eGFP-PoPit, pSK35S-p61:eGFP-PoPit, pSK35S-MP:eGFP-PoPit and pSK35S-p24:eGFP-PoPit, contained the corresponding CiLV-C gene fused to the eGFP under the control of 35S constitutive promoter from cauliflower mosaic virus (CaMV) and the terminator from the potato proteinase inhibitor (PoPit). Then, the correspondent 35S-(CiLV-C genes):eGFP expression cassettes were subcloned into the pMOG_800_ binary vector by using the restriction sites *Hind*III (for *p15*), *EcoR*I/*Xho*I (for *p29, MP*, and *p24*) and *EcoR*I/*Sac*I (for *p61*). For N terminal fusion, the genes were cloned into the vector pSK35S-eGFP:GFP-PoPit replacing the GFP gene and subcloned into the pMOG800 binary vector, such as aforementioned.

For the Bimolecular Fluorescence Complementation (BiFC), membrane topology and *in vivo* viral protein-protein interaction assays, we fused the N-terminal 154 amino acids of the yellow fluorescent protein sequence (N-YFP) or the C-terminal 84 amino acids of the YFP (C-YFP) to the N or C terminus of all CiLV-C proteins. Detailed handling for obtaining the pSK35S-N-YFP:eGFP-PoPit, pSK35S-C-YFP:eGFP-PoPit, pSK35S-eGFP:N-YFP-PoPit, and pSK35S-eGFP:C-YFP-PoPit constructs was previously described (Leastro et al., 2015). For fusing the N-YFP or C-YFP at the N- and C-terminus of CiLV-C *p29, p15, p61, MP*, and *p24*, the eGFP gene was replaced using the respective restriction sites aforementioned. The expression cassettes containing the CiLV-C genes were subcloned into the pMOG_800_ binary vector.

The constructions, which contained the N- and C-terminal fragments of the EYFP addressed to the ER lumen (N-YFPer and C-YFPer) and cytosol (N-YFPcyt and C-YFPcyt) were provided by Dr. Jari P.T Valkonen, University Helsinki (Zamyatnin et al., 2006) and by Dr. F. Aparicio; Instituto de Biología Molecular y Celular de Plantas “Pirmo Yúfera,” Valencia/Spain (Aparicio et al., 2006), respectively. The first one-cassette constructions (N-YFPer and C-YFPer) were obtained from pRT-YN-ER and pRT-YC-ER vectors (Zamyatnin et al., 2006) and subcloned into the binary vector pMOG_800_. All DNA manipulations were confirmed by plasmid DNA sequencing.

The ER (mCherry-HDEL) and Golgi (Man49-mCherry) markers (Nelson et al., 2007) were obtained from the University of Tennessee (Knoxville/USA). The Actin marker (dsRFP-Talin) (Genovés et al., 2009), HA-tagged Lep, HA-tagged NSm and TSWV (N/NSm) BiFC constructs were provided by Dr. J.A. Sánchez-Navarro, Instituto de Biología Molecular y Celular de Plantas “Pirmo Yúfera,” Valencia/Spain.

### Intracellular distribution and Co-localization of CiLV-C proteins, *in vivo*

To investigate the intracellular distributions of CiLV-C proteins in plant cells, *Agrobacterium tumefaciens* (strain C58C1) containing the viral genes fused at their N- or C-termini with eGFP in pMOG_800_ binary vector was infiltrated in *N. benthamiana* leaves (OD_600_ = 0.4) as described previously (Leastro et al., 2015).

To visualize the co-localization of the CiLV-C proteins with ER, Golgi, and actin organelles, simultaneous expression of two proteins in individual bacteria cultures containing the correspondent binary vectors was performed. For cultures of *A. tumefaciens* transformed with organelle markers in binary vector, OD_600_ was adjusted to 0.5 and mixed with the respective agrobacteria containing the CiLV-C genes, before leaf infiltration. The plants were maintained in FITOTRON® plant growth chamber under conditions of 23°C day 18°C night, 70% humidity and 16 h light / 8 h dark regime. The fluorescence was monitored at 72 h post-infiltration (hpi), with the exception of p61, whose fluorescence expression was determined at 24-36 hpi.

For callose staining, *N. benthamiana* leaves were infiltrated with aniline blue (Merk, Darmstadt, Germany) solution at 0.005% concentration in sodium phosphate buffer, 70 mM, pH 9.0. The leaves were infiltrated and kept in a dark room 20 min before confocal visualization.

All confocal images of co-localization were obtained after repeated visualization of different cells and regions of the *N. benthamiana* transiently transformed leaves.

### Bimolecular fluorescence complementation assays

In BiFC assays, *A. tumefaciens* (strain C58C1) cultures (OD_600_ = 0.4) transformed with the corresponding binary plasmid pMOG_800_ were used to infiltrate *N. benthamiana* plants as previously described (Genovés et al., 2011). The plants were kept at 23°C day 18°C night, 70% humidity with 16 h light / 8 h photoperiod. At 4 days post-infiltration, the fluorescence reconstitution was observed.

For the BiFC assay, addressed to characterize the CiLV-C proteins topology, the *p29, p15, MP*, and *p24* carrying the N-YFP or C-YFP fused at the N- or C-terminus were transiently expressed with the counterpart addressed to cytosol (N-YFPcyt and C-YFPcyt) or ER lumen (C-YFRer and N-YFPer). This technique relies on the capability of two non-fluorescent fragments of the EYFP, the N- (N-YFP, 1-154 amino acids) and C-termini (C-YFP, 155-239 amino acids), to interact with each other when they are overexpressed in the same subcellular compartment (Zamyatnin et al., 2006). All protein pair combinations preformed in the co-infiltration are shown in Table 3. To increase the expression, in order to allow a better visualization of the fluorescence signal, all protein pair combinations were co-expressed with the silencing suppressor HcPro from tobacco etch virus.

For *in vivo* protein-protein interaction, all CiLV-C proteins were fused at their N- and C- termini with N-YFP and C-YFP. In dimerization analysis, performed individually for each CiLV-C protein, the indicated pair of proteins was transiently expressed in *N. benthamiana* as described above. The same was performed in the heterodimerization association; however, the combination pairs were performed between different CiLV-C proteins. Tables 1, 2 summarize all combinations performed in dimerization and heterodimerization assays, respectively.

**Table 1:** Synthesis of all protein pair combinations performed in the BiFC dimerization assay.

**Dimers**	**p29**	**p15**	**p61**	**MP**	**p24**
ORF-NYFP + CYFP-ORF	+++	++	–	–	–
ORF-NYFP + ORF-CYFP	+++	+++	–	–	+++
NYFP-ORF + CYFP-ORF	–	+	–	–	–
NYFP-ORF + ORF-CYFP	+	+++	–	–	–
**NEGATIVE CONTROLS**				
CYFP-ORF+NYFP-Cyt	–	–	–	–	–
ORF-CYFP+NYFP-Cyt	–	–	–	–	–
NYFP-ORF+CYFP-ER	–	–	–	–	–

*The symbols –, +, ++, and +++ correspond to the absence, low, medium or high fluorescence signals, respectively. NtYFP and CtYFP correspond to half N- and C-terminal of the YFP protein, respectively*.

**Table 2:** Synthesis of all protein pair combinations performed in the BiFC hetero-dimerization assay.

**Heterodimers**	**p29-p15**	**p29-MP**	**p29-p24**	**p15-MP**	**p15-p24**	**MP-p24**
ORF-NYFP + CYFP-ORF	+++	+	–	–	–	–
ORF-NYFP + ORF-CYFP	+++	+	–	++	–	–
NYFP-ORF + CYFP-ORF	+	–	–	–	–	–
NYFP-ORF + ORF-CYFP	–	–	–	–	–	–
ORF-CYFP + NYFP-ORF	+++	+	++	–	–	–
ORF-CYFP + ORF-NYFP	+++	+++	–	+++	–	–
CYFP-ORF + NYFP-ORF	++	+	–	–	–	–
CYFP-ORF + ORF-NYFP	+++	++	–	+	–	–
Negative controls	p29	p15	MP	p24		
CYFP-ORF+NYFP-Cyt	–	–	–	–		
ORF-CYFP+NYFP-Cyt	–	–	–	–		
NYFP-ORF+CYFP-ER	–	–	–	–		

*The symbols –, +, ++, and +++ correspond to the absence, low, medium or high fluorescence signals, respectively. NtYFP and CtYFP correspond to half N- and C-terminal of the YFP protein, respectively*.

### Confocal laser scanning microscopy

Fluorescence images were captured with the aid of a confocal laser scanning microscope Leica TCS SP8 model. GFP fusion proteins were excited at 488 nm and emission was captured at 495–520 nm. YFP was excited at 514 nm and emission was captured at 520–560 nm. The dsRFP and mCherry fluorophores were excited at 552 nm and emission was captured at 585–610 nm. Chlorophylls were excited at 488 nm and emission was captured at 660–720 nm. Analine blue was excited at 405 nm and emission was captured at 410–480 nm. Time-lapse images and videos were taken using the Leica X microscope software. Each frame was obtained every 10 s and a total of approximately 50 frames were obtained for each video. The videos were edited to play eight frames per second using image J program (version 1.46r). As aid of the LAS AF quantify intensity tool were generated the graphs of fluorescence intensity for a better interpretation of the co-localization. Standard deviation in Person correlation coefficient (PCC) was measured using the Fuji co-localization plugin from three or four independent images. In general, PCC values of 0.2–0.4 indicate weak positive correlations, values above 0.5 indicate strong positive correlations and negative values indicate opposite correlation (Dunn et al., 2011; Ishikawa et al., 2017).

### CiLV-C proteins expression, membrane sedimentation and western blot analyses

*A. tumefaciens* strain C58C1 cultures were transformed with binary pMOG_800_ plasmid, containing the *p29, p15, MP*, and *p24* genes fused at their C-terminal to the eGFP or HA-tagged for Lep and NSm proteins. Agrobacterium strain GV3101 was transformed with the pUC35s-GFP-HDEL plasmid that expresses free GFP with ER retention signal. The cultures (OD_600_ = 0.4) were individually agroinfiltrated in *N. benthamiana* leaves as referred above. After 3 dpi, the leaves were processed to obtain enriched membranous fraction. Approximately 1.5 g of infiltrated leaf was ground in lysis buffer (20 mM HEPES [pH 6.8], 150 mM potassium acetate, 250 mM mannitol, 1 mM MgCl_2_ and 50 μL of protease inhibitor cocktail for plant cell, Sigma), and the homogenate was clarified by centrifugation at 3,000 g for 10 min at 4°C. The collected supernatant was ultracentrifuged at 40,000 g for 40 min at 4°C to yield the soluble (_1_S30) and the crude (_1_P30) microsomal fractions. Microsomal pellets were resuspended in lysis buffer and divided into four fractions, to which the same volume of original lysis buffer were added (control aliquot), 100 mM Na_2_Co_3_ (pH 11.0), 4M and 8M urea and incubated for 30 min on ice. The supernatant fractions (_2_S30) were collected by ultracentrifugation at 40,000 g for 40 min at 4°C and the respective pellets (_2_P30) were resupended in the same volume with lysis buffer. All the fractions were analyzed by Western blot in 12% SDS-PAGE gels. The gel was electrotransferred to polyvinylidene difluoride membranes following the manufacturer's instructions (Amersham). The detection of the proteins tagged with GFP or HA was performed by using an N-terminal GFP antibody (Sigma), an anti-HA antibody (Fermentas^TM^), and a secondary antibody conjugated with the peroxidase (Sigma). The chemiluminescence detection was made using the substrate recommended by Sigma (Tetramethylbenzidine [TMB], liquid substrate system for membrane).

Percentage values of relative concentration of CiLV-C proteins associated with membrane or released after chemical treatments were measured with the program Quant TL (8.1.0.0). Reference value equivalent to 100% was given based on the pixels quantification of the controls treated with lysis buffer.

### Prediction analyses

Transmembrane and hydrophobic regions from residues of the CiLV-C proteins were predicted with different algorithms using the TMHMM server (http://www.cbs.dtu.dk/services/TMHMM/), Phorbius (http://phobius.sbc.su.se Kall et al., 2007) and MPEx (Snider et al., 2009). Coiled coil structures were predicted with PCOILS server (Alva et al., 2016).

For *p15*, nuclear localization signal (NLS) and nuclear exportation signals (NES) were tentatively predicted with NLS mapper [nls-mapper.iab.keio.ac.jp (Kosugi et al., 2009)] and NetNES server [www.cbs.dtu.dk (la Cour et al., 2004)], respectively.

For p61, the presence of signal peptide was predicted with SignalP 4.1 server [cbs.dtu.dk/services/SignalP/ (Petersen et al., 2011)] and N-glycosylation with NetNGlyc 1.0 server (cbs.dtu.dk/services/NetNGlyc/).

CiLV-C *p29, p15, p61, MP*, and *p24* ORFs used in this study were sequenced and the predicted proteins exhibited 100% identity with those available in the GenBank accession numbers YP_654539.1, YP_654540.1, YP_654541.1, YP_654542.1, and YP_654543.1, respectively).

## Results

### Cytopathic effects from CiLV-C infection are mainly associated with ER disorders

**Figure 1:**
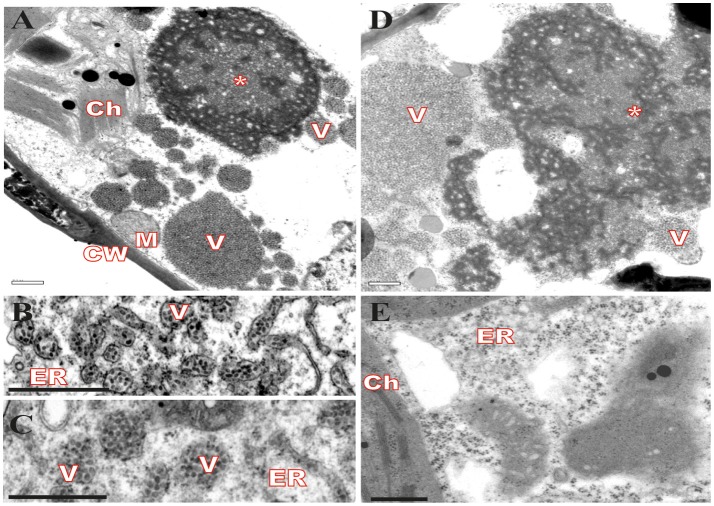
Transmission electron micrographs from local lesion on leaves of plants experimentally inoculated by *Brevipalpus yothersi* viruliferous for CiLV-C shows ER membrane disorder. **(A)** *Glycine max*, **(B)** *Abelmoschus esculentus*, **(C)** *Nicotiana clevelandii*, **(D)** *Portulaca oleracea* and **(E)** non-infected *G. max*. ER membrane remodeling is observed in all infected cells, more evident in **B** and **C**, with the formation of vesicles comprising short-bacilliform (55–70 nm × 100–110 nm) CiLV-C particles. Characteristic electro-dense viroplasm in the ER lumen on cytoplasm is observed in **A** and **D**. Viroplasm (*), CiLV-C particle (V), Endoplasmic reticulum (ER), Cell wall (CW), Chloroplasts (Ch), and Mitochondria (M). Bars correspond to 0.5 μm.

Virus infection usually causes changes in host plant cells resulting in morphological and metabolic disturbances. In general, accumulation of viral proteins and alteration in cell endomembrane system associated with viral replication and assembly are noticeable (Lesemann, 1991; Lawson et al., 1996; Laliberté and Sanfaçon, 2010). Cytopathic effects resulting from CiLV-C infection were investigated in leaves of plants experimentally inoculated with viruliferous *B. yothersi* mites. After leaf inoculation, small fragments of these lesions were processed and examined by transmission electron microscopy (TEM). In all of the assessed hosts, *Nicotiana clevelandii, Abelmoschus esculentus, Portulaca oleracea*, and *Glycine max*, changes in ER complex were observed, with the formation of vesicles containing shot-bacilliform virions in their cisternae (Figures 1B,C) and the appearance of characteristic electron-dense vacuolated viroplasm in the cytoplasm (Figures 1A,D). The same cytopathic effects were observed in several other species of plants infected by CiLV-C (Garita et al., 2014), suggesting that regardless of the host, the process of viral infection results in virion accumulation in ER cisternae, possibly resulting from a budding process of viral morphogenesis on ER membranes. In some occasions, CiLV-C virions were present in the perinuclear cavity, which is considered an extension of the ER (data not shown). Figure 1E shows an area of an uninfected plant lef (*Glycine max*), where the aforementioned pattern was not observed.

### Subcellular localization of CiLV-C proteins in plant cells

**Figure 2:**
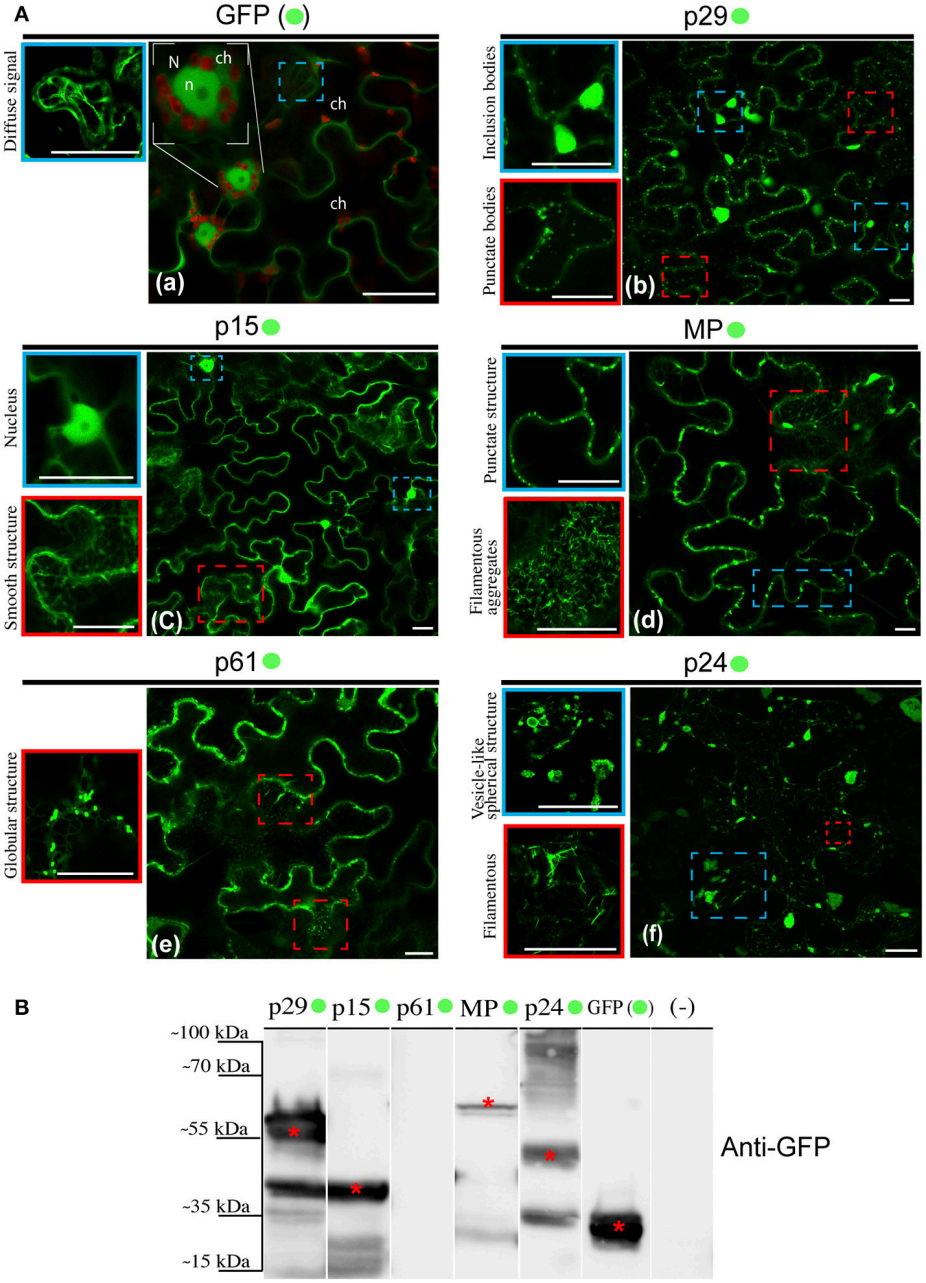
Structure formation and intracellular distribution from transient expression of CiLV-C proteins fused with eGFP and unfused eGFP. **(A)** Localization analyses from expression of the p29, p15, p61, MP, and p24 CiLV-C proteins fused C-terminal with eGFP (•) in epidermal cell of *N. benthamiana*. The images shows: **(a)** Unfused eGFP, which generates a diffuse signal evenly distributed in the cytoplasm (blue box). The nucleolus (n) is visualized internally to the nucleus (N), and auto-fluorescence is observed in the chloroplasts (ch). **(b)** The presence of p29:eGFP punctate bodies (red box) and inclusion bodies (blue box) dispersed throughout the cytoplasm. **(c)** The p15:eGFP apparently is organized in a smooth structure that aligns with structures similar to the ER network (red box) and into the nucleus (blue box). **(d)** The MP:eGFP punctate structures localized at the cellular periphery (blue box), and numerous filamentous aggregates where observed when focused on the cytoplasm region (red box). **(e)** The p61:eGFP in punctate bodies apparently associated with structures similar to the ER. **(f)** Formation of vesicle-like spherical (blue box) and filamentous structures (red box) from p24:eGFP expression in the cytoplasm. Images on the left show in higher magnification the respective structures indicated in the blue or red dashed boxes. Fluorescence signals were captured 72 h post-infiltration (except for p61, captured 24–36 hpi) with confocal microscope Leica SP8 model. White bars correspond to 50 μm. **(B)** Western blot analysis of the transient accumulation of GFP (~27 kDa), p29-•(~56 kDa), p15-•(~42 kDa), MP-•(~59 kDa), and p24-•(~51 kDa) proteins in *N. benthamiana* leaves. Labeled protein was not detected from p61-•(~88 kDa) transient expression. Monoclonal antibody against GFP (Sigma) was utilized to detect the CiLV-C proteins fused to the eGFP and unfused eGFP, which are indicated with*. (–) corresponds to a non-agroinfiltrated plant.

Given the ER modifications observed upon CiLV-C infection, we asked what would be the direct involvement and effect of each CiLV-C protein in cytopathic changes, since there are no reports on their functionality. In this context, we first investigated the formation of structures from transient expression of CiLV-C proteins fused with eGFP (enhanced green fluorescent protein) and their intracellular distribution in the cell. To this end, *N. benthamiana* leaves were agroinfiltrated with the binary constructs containing the p29, p15, MP, p61, or p24 fused with eGFP at their C-terminal and the GFP signal was visualized for each situation. Compared with unfused eGFP, which generates a diffuse signal evenly distributed in the cytoplasm and internally to the nucleus (Figure 2Aa), the p29:eGFP was organized in two distinct patterns: in numerous fluorescent punctate bodies, and in large inclusion bodies dispersed throughout the cytoplasm (Figure 2Ab). The p15:eGFP did not show a specific format and seems to be organized as a smooth structure that aligns well with structures similar to the ER network. Z-stack microscopy images indicated that among all CiLV-C proteins, only p15 occurs intranuclearly (Figure 2Ac), where its distribution and location is apparently similar to unfused GFP expression in plant cells. The MP:eGFP was initially viewed in small punctate structure along the plasma membrane (PM) in epidermal cells, suggesting a possible association with PD. Furthermore, numerous filamentous aggregates were observed in the cytoplasm (Figure 2Ad). The p61:eGFP was organized in globular structures dispersed throughout the cytoplasm, where apparently was associated with ER network (Figure 2Ae). This structure was also organized in agglomerates (data not show). Numerous filamentous and vesicle-like structures dispersed in the cytoplasm were visualized from p24:eGFP expression (Figure 2Af). In order to confirm the fused proteins expression, a Western blot was performed with specific antibody against GFP. Band detection of the expected size was observed for all proteins, expect for p61:eGFP (Figure 2B). Intriguingly, the amount of cells expressing fluorescence signal for this construction is visually lower than that observed for the other CiLV-C fused proteins (data not shown). In addition, approximately 36–48 h after p61 infiltration in plant leaves, it clearly induced necrosis formation on the infiltrated tissue (Figure S1), suggesting that this protein triggers some process of plant defense that causes cell death.

A limitation in sub-localization assays by using fluorescent fusion proteins relies on the possible interference in folding of the target protein that may result in changes in its location and functionality. For this reason, the five CiLV-C proteins, in addition to their expression with the extreme free N-termini (assay aforementioned), were also expressed containing a free C-termini (N- attached). To do this, the eGFP was fused at the N-terminus of each protein and visualized as previously reported. The localization of fluorescence signals from p29, p15, and MP proteins fused to the eGFP were similar at both termini (Figures S2a–c, respectively). Expression of eGFP:p24 showed association with structures similar to the ER network with the formation of filamentous structures. However, differently from the p24:eGFP fusion, the formation of vesicular structures were not observed (Figure S2d). Expression of eGFP:p61 yielded low fluorescence return in a low number of cells (Figure S2e). These results suggest that for p29, p15, and MP, regardless of the fusion region (N- or C-terminal), there are no changes in their intracellular localization. In contrast, the p24 apparently underwent changes in functionality by fusing the N-terminal, and for p61 the fluorescence was not detected. Based on these findings, for the subsequent assay of co-localization with cell organelles, we chose to use the constructions with free N-termini.

### CiLV-C proteins associate with ER and p15, p61, and p24 cause disturbance in the ER network

**Figure 3:**
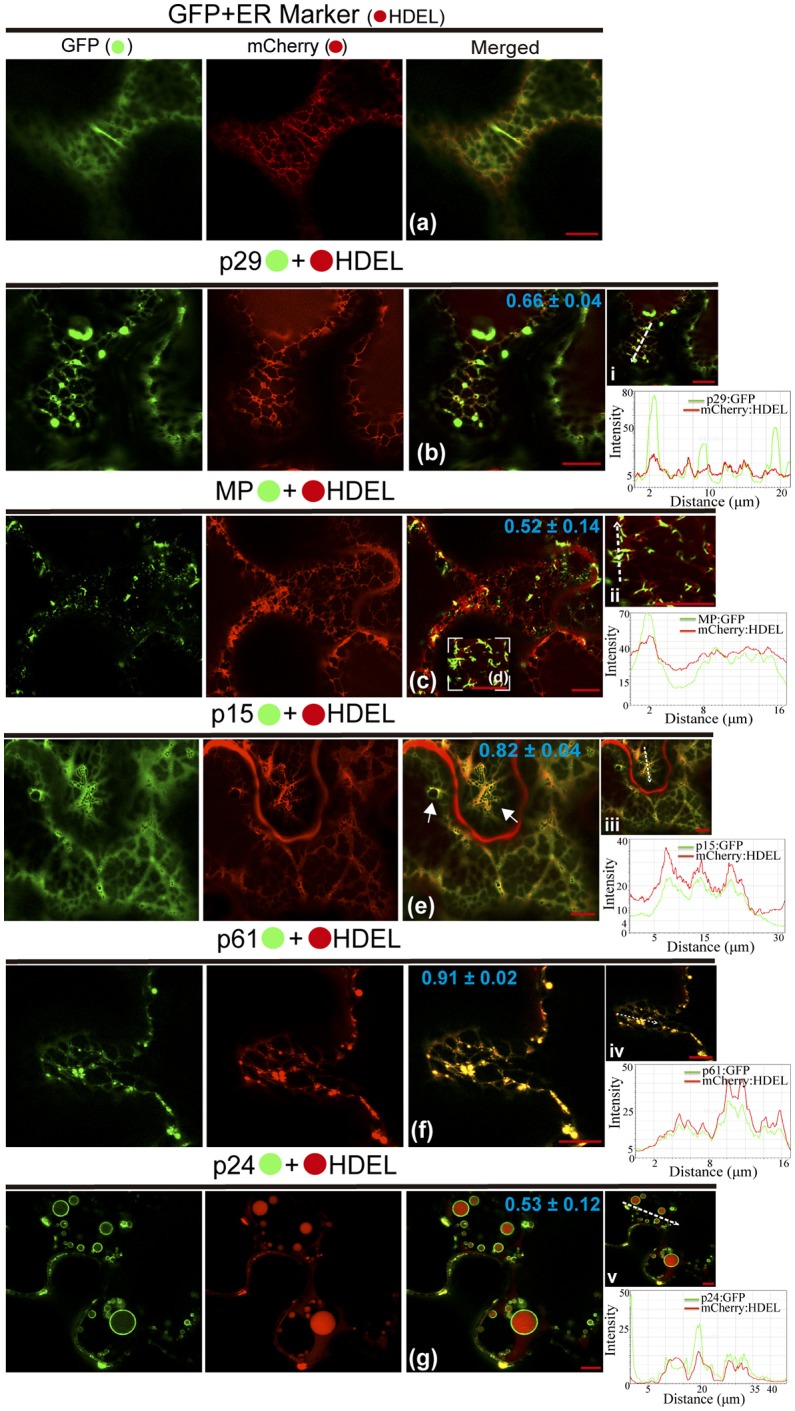
CiLV-C proteins associate with ER and p15, p61, and p24 remodel ER network. The p29, p15, p61, MP, and p24 proteins fused at their C-termini with eGFP (•) were co-expressed with mCherry(•)-HDEL (ER marker) in epidermal cells of *N. benthamiana*. Fluorescence signals were captured 72 h post-infiltration (except for p61, captured 24–36 hpi) with confocal microscope Leica SP8 model. The green (GFP), red (mCherry) channels and merged images are shown for each CiLV-C protein expressed with ER marker. **(a)** Image shows unfused GFP expression in a diffuse signal evenly distributed in the cytoplasm, where an intact ER network is observed. **(b)** Image shows p29:eGFP expression in punctate bodies co-localizing with ER. **(c,d)** Co-localization of the MP:eGFP filamentous aggregates with ER network, without apparent ER changes. **(e)** ER remodeling is observed from expression of the p15:eGFP with the formation of pleomorphic structures (arrows) throughout the ER cisternae. **(f)** Co-localization of the p61:eGFP and ER showing deformation in the ER cisternae with the formation of globular bodies along the ER. **(g)** Merged image shows that p24:eGFP expression completely remodel the ER, reorganizing it in vesicle-like spherical structures surrounded by the protein. The mean *SD* of Person Correlation Coefficient (PCC) is given in the merged image. Intensity profiles along the line segments, shown in the images on the right and represented by graphs, demonstrate positive co-localization in i, ii, iii, iv, and v. Plot shows green and red fluorescence intensities, indicated by (CiLV-C-Proteins):GFP and ER:mCherry, in the selected region of interest (dotted arrows). Distance measurement starts from the base to the tip of the arrows (x axis). Fluorescence intensity graphs were generated using Leica LAS AF quantify intensity tool. Red bar correspond to 10.

In order to dissect the subcellular structures involved with the localization pattern aforementioned and to provide further evidence of functionality of the proteins, we investigated the association of proteins with ER, GC, and actin. These organelles were chosen, since most plant viruses use them for their replication and spread (Heinlein, 2015; Kleinow, 2016). In a first step, we analyzed the proteins association with ER network from co-expression of each protein containing the GFP fused with the ER marker mCherry-HDEL. When the unfused eGFP and mCherry-HDEL marker were co-expressed, a diffuse signal evenly distributed in the cytoplasm (showed in Figure 2) aligned with the ER network, but no modification in the ER was observed (Figure 3a). The co-expression of p29:eGFP and mCherry-HDEL marker revealed a co-localization of the protein and the ER (Person correlation coefficient [PCC] = 0.66 ± 0.04), through the appearance of p29:eGFP punctate bodies along the ER cisternae, without causing structural modification to the network (Figure 3b). p29:eGFP inclusions bodies also were visualized along the ER (see Movie S3). The filamentous aggregates formed from MP:eGFP expression were associated with the ER (PCC = 0.52 ± 0.14), also without causing structural modifications (Figures 3c,d). However, the same pattern of integrity in the cortical ER structure was not observed after the expression of p15:eGFP, p61:eGFP, and p24:eGFP proteins. The p15:eGFP completely co-localized with ER (PCC = 0.82 ± 0.04) forming a smooth structure that aligned with the ER network, causing its disruption (Figure 3e, arrows). The co-expression of ER marker and p61:eGFP revealed a strong co-localization (PCC = 0.91 ± 0.02), and the globular structures generated disruption of the organelle (Figure 3f). Interestingly, p24:eGFP completely remodeled the ER, reorganizing it in vesicle-like spherical structures completely surrounded by viral protein (PCC = 0.53 ± 0.12; Figure 3g). Co-localization of the CiLV-C proteins with ER was further analyzed by measurement of fluorescence intensity across the ER. Fluorescence signals of p29-eGFP, MP-eGFP, p15-eGFP, p61-eGFP, and p24-eGFP coincide with the ER marker signal, showing that all proteins were localized at the ER (Figures 3i–v, respectively).

### All proteins (except p29) co-localize with GC and remodel the distribution of the golgi apparatus

**Figure 4:**
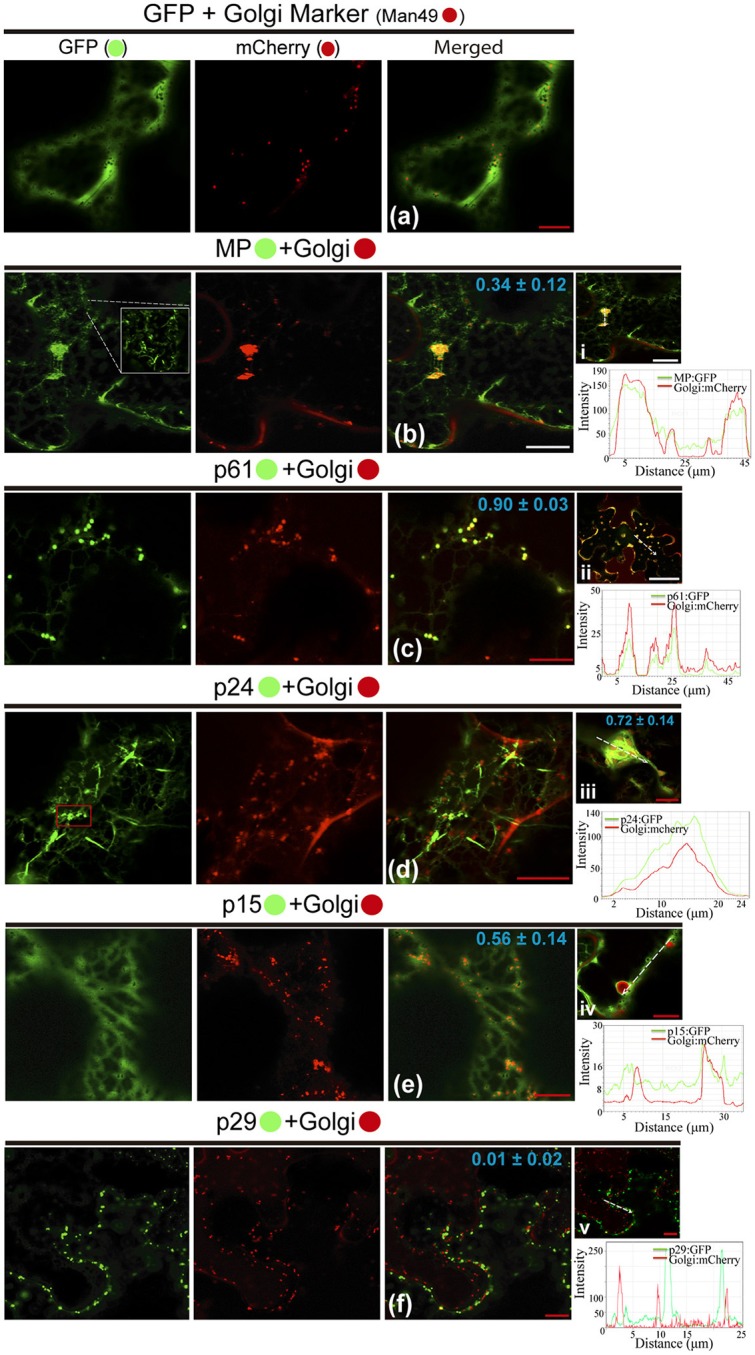
All CiLV-C proteins (except p29) co-localize with Golgi and remodel the distribution of the GC apparatus. The p29, p15, p61, MP, and p24 proteins fused at their C-termini with eGFP (•) were co-expressed with man49-(•)mCherry (Golgi marker) in epidermal cells of *N. benthamiana*. Fluorescence signals were captured 72 h post-infiltration (except for p61, captured 24–36 hpi) with confocal microscope Leica SP8 model. The green (GFP), red (mCherry) channels, and merged images are shown in the figure. **(a)** Image exhibits unfused GFP expression in a diffuse signal evenly distributed in the cytoplasm with individualized GC circular structures also dispersed throughout the cytoplasm. **(b)** Merged image shows that MP:eGFP and Golgi co-localize, with MP:eGFP filaments intertwined the Golgi apparatus, forming pleomorphic agglomerates in the cytosol. White box in the GFP channel shows in more detail the MP filaments. **(c)** Co-localization of the p61:eGFP globular bodies with Golgi apparatus. (ii) The image shows the aggregates formed from association of the p61:eGFP and GC dispersed throughout the cytoplasm. **(d)** The p24:eGFP filaments and spherical structures (red box) are observed merged with Golgi apparatus. (iii) Curious cytopathic effect of the p24:eGFP expression, with a remodeling of Golgi apparatus in flattened circular aggregates, with the viral protein, in long parallel filaments covered the GC aggregates forming inclusions. **(e)** p15:eGFP co-localizes with GC, with the protein apparently associated with ER, which emerges on Golgi apparatus. (iv) Merged image exhibits changes in the GC distribution with the formation of circular Golgi aggregates internalized possibly by ER containing the p15:eGFP proteins diffused along the ER network. **(f)** No co-localization of the p29:eGFP punctate bodies with GC. The mean *SD* of PCC at the top of the merged image, and chart of fluorescence intensities further confirm the co-localization of the MP, p61, p24, and p15 with Golgi (merged pictures i–iv, respectively). Plot shows green and red fluorescence intensities, indicated by (CiLV-C-Proteins):GFP and Golgi:mCherry, in the selected region of interest (dotted arrows). Distance measurement starts from the base to the tip of the arrows (x axis). Red and white bars correspond to 10 and 50 μm, respectively.

Possible association of CiLV-C encoded proteins with Golgi complex and subsequent changes in it was studied by the co-expression of all viral proteins fused to the eGFP and Man49-mCherry (GC marker) in leaf epidermal cells. The co-expression of unfused eGFP and GC marker did not change the distribution of the Golgi apparatus, and the GFP showed a diffuse signal (Figure 4a). For MP:eGFP, the protein organized in filaments (see the increased image, Figure 4b) was intertwined with the Golgi apparatus, and GC remodeling in the form of inclusions was observed in the cytosol (PCC = 0.34 ± 0.12; Figure 4b). Co-expression of the p61:eGFP with GC marker resulted in the co-localization of the globular structures of the p61:eGFP complex with Golgi bodies (PCC = 0.90 ± 0.03, Figure 4c), forming numerous inclusions dispersed throughout the cytoplasm (Figure 4ii). For p24:eGFP co-expressed with GC marker, small spherical structures (possibly vesicles, see red box in GFP channel) and flexuous filaments, loosely scattered in the cytoplasm, were observed (Figure 4d). Furthermore, we observed the co-localization with a remodeling of Golgi apparatus in flattened circular aggregates, where the p24:eGFP, in long parallel filaments, covered the GC aggregates forming inclusions (p24:eGFP-Golgi) (PCC = 0.72 ± 0.14; Figure 4iii). The p15:eGFP apparently associated to the ER fuses with Golgi apparatus (Figure 4e) and modifies the GC with the formation of circular aggregates internalized by possible p15:eGFP-ER network (PCC = 0.56 ± 0.14; Figure 4iv). Differently from the other proteins, p29:eGFP punctuated bodies did not co-localize with GC (PCC = 0.01 ± 0.02; Figure 4f). Measurements of fluorescence intensity across the Golgi show that fluorescence signals of MP:eGFP, p61:eGFP, p24:eGFP, and p15:eGFP coincide with the Golgi complex marker signal, showing that these proteins co-localized with GC (Figures 4i–iv). However, p29 GFP fluorescence signal poorly coincide with the Golgi signal (Figure 4v), suggesting that they did not co-localize.

### The p29 and p24 proteins are associated with actin, and MP punctate structures co-localize with callose at the cell periphery

**Figure 5:**
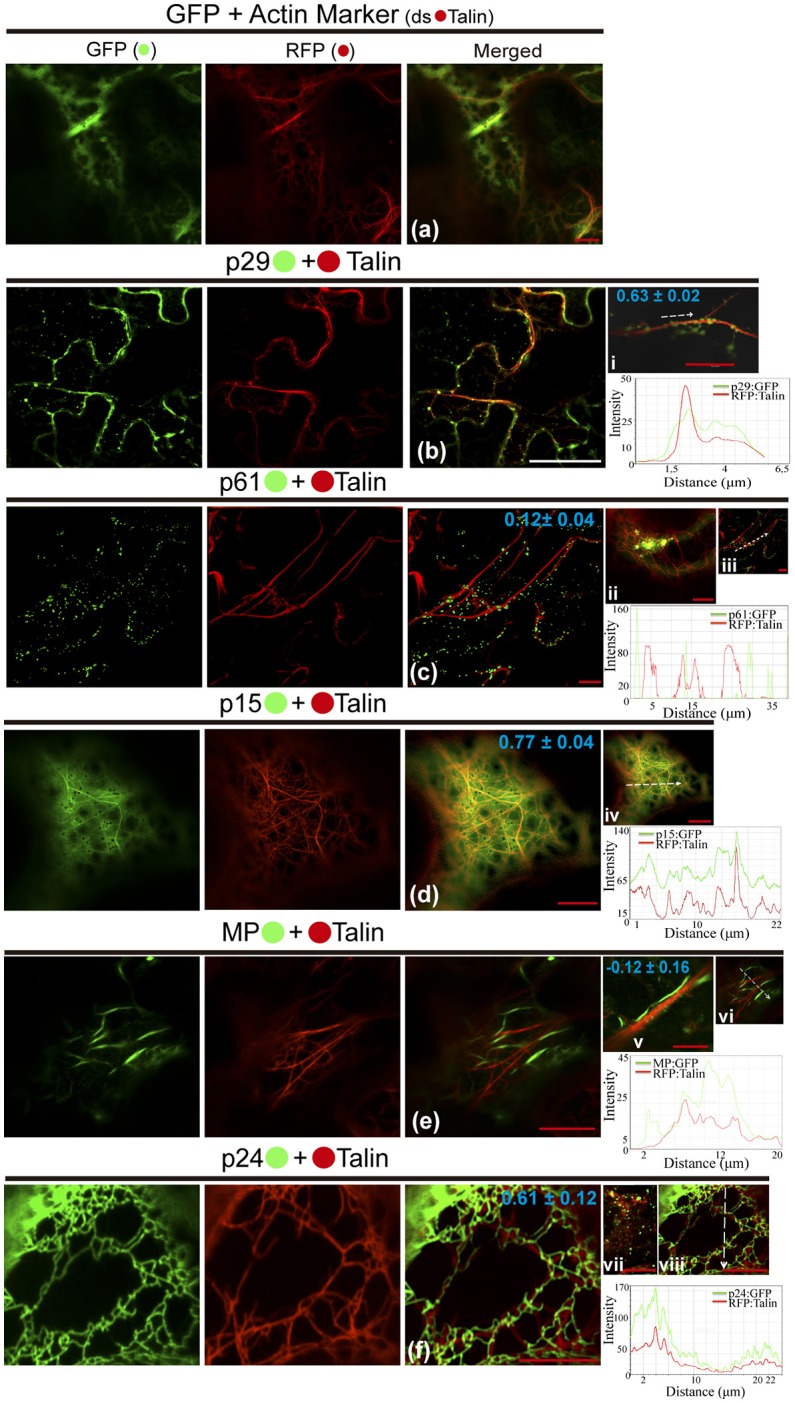
The p29, p15, and p24 CiLV-C proteins co-localize with actin microfilaments. The p29, p15, p61, MP, and p24 proteins fused at their C-termini with eGFP (•) were co-expressed with dsRFP-(•) Talin (actin marker) in epidermal cells of *N. benthamiana*. Fluorescent signals were captured 72 h post-infiltration (except for p61, captured 24–36 hpi) with confocal microscope Leica SP8 model. The green (GFP), red (RFP) channels and merged images are shown in the figure. **(a)** Image show unfused GFP expression in a diffuse signal evenly distributed in the cytoplasm with tagging of actin skeleton from expression of the actin marker. **(b)** Epidermal cells show p29:eGFP punctuate bodies associated with actin filaments. Image in (i) shows only one actin filament with several p29:eGFP punctate bodies co-localized along the filament. **(c)** No co-localization of the p61:eGFP individual bodies or agglomerates (ii) along the actin filaments. **(d)** p15:eGFP, apparently diffused throughout the ER, co-localizes emerging on actin network. **(e)** Image shows MP:eGFP filaments expression that do not co-localize with actin marker. This observation is more conclusive in image (v), where direct association between the MP:eGFP filaments along the actin filament is not visualized in a higher magnification. **(f)** Epidermal cells showing actin filaments wrapped around by p24:eGFP filament-like structures. (vii) Image of the p24:eGFP in spherical structures (possible vesicles) is observed throughout the actin filaments. The mean SD of PCC at the top of the merged image and chart of fluorescence intensities further confirm the co-localization of the p29, p15, and p24 with actin (merged pictures i, iv, viii, respectively). Plot shows green and red fluorescence intensities, indicated by (CiLV-C-Proteins):GFP and RFP:Talin in the selected region of interest (dotted arrows). Distance measurement starts from the base to the tip of the arrows (x axis). Red and white bars correspond to 10 and 50 μm, respectively.

We also investigated the involvement of the CiLV-C proteins with cytoskeleton through the visualization of their co-expression with dsRFP-Talin (actin marker). The co-expression of unfused eGFP and actin marker showed a diffuse signal in the cytoplasm (Figure 5a), such as visualized by all previous situations of the controls. The p29:eGFP punctate bodies were localized along the actin filaments (PCC = 0.63 ± 0.02; Figure 5bi). For the p61 protein, the p61:eGFP globular structures along the actin filaments (PCC = 0.12 ± 0.04) were absent, and the same pattern was observed when the globular structures were organized in agglomerates (Figure 5cii, respectively), suggesting absence of interaction between p61 and actin. Over again, the same expression phenotype of p15:eGFP previously identified was observed. This protein was apparently aligned with ER, which was intermingled with actin filaments (PCC = 0.77 ± 0.04; Figure 5d). The MP:eGFP filaments apparently did not co-localize with the actin filaments (Figure 5e). To better clarify this, more detailed images from the expression of MP:eGFP close to actin filaments were taken and did not reveal interaction between MP and actin (PCC = −0.12 ± 0.16; Figure 5v). The filamentous form of p24:eGFP and actin were co-localized (PCC = 0.61 ± 0.12), with the protein complex wrapped around the actin filaments (Figure 5f). Small vesicle-like structures of p24:eGFP were also visualized (Figure 5vii). Rearrangement of the microfilaments was not observed, in contrast to what was seen when the same CiLV-C proteins were associated with ER and GC. Measurements of fluorescence intensity across the actin showed that fluorescence signals of p29:eGFP, p15:eGFP, and p24:eGFP coincide with those of the actin marker signals (Figures 5i,vi,viii, respectively), strengthening the likely co-localization of these proteins and actin filaments. On the other hand, p61:eGFP and MP:eGFP fluorescence signals did not coincide with mCherry signal along the actin filament (Figures 5iii,vi, respectively), suggesting that these proteins did not co-localize with actin.

Given that viral movement proteins have the capability to associate with plasmodesmata ensuring the cell-to-cell viral movement, and observing the punctuated structures at cell peripheries formed by MP:eGFP expression, we investigated this possible association by the infiltration of fluorochrome aniline blue (callose staining) on *N. benthamiana* leaves expressing the MP protein. It was clearly observed that MP punctate structures co-localize with callose deposits at the cell periphery (Figure S3), suggesting its association with PD.

Co-localization assays clearly demonstrated the localization of CiLV-C proteins with ER (p29, p15, p61, MP, and p24), GC (p15, MP, p61, and p24), microfilaments (p29, p15, and p24), nucleus (p15) and plasmodesmata (MP) *in vivo*; clarifying better the mentioned structures initially observed from the individual expression of CiLV-C proteins fused with eGFP. Furthermore, we observed unprecedented cytopathic effects in the remodeling of ER and GC structures following the interaction with viral proteins.

### The p29 and MP proteins trafficking along the ER system

**Figure 6:**
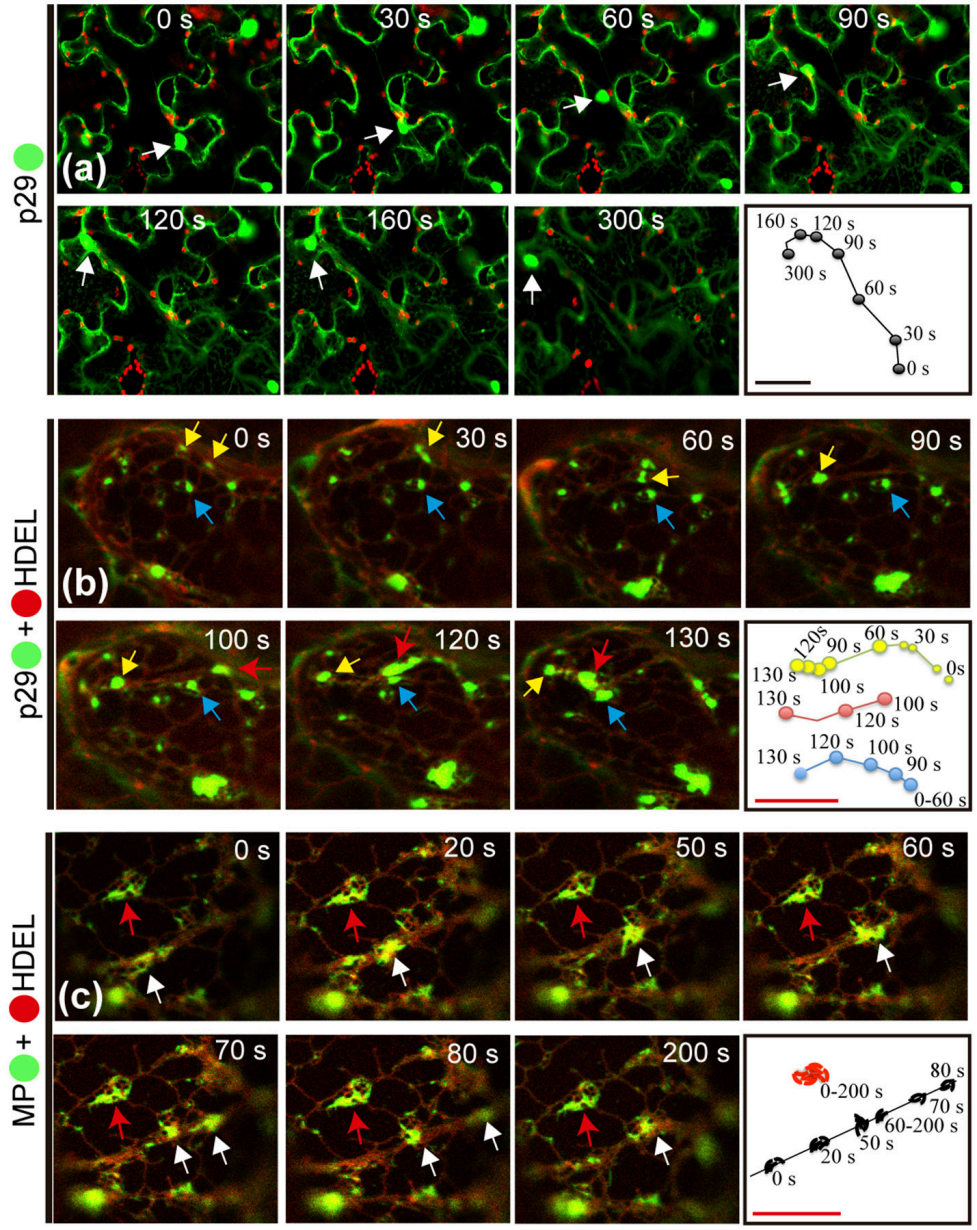
Citrus leprosis virus C p29 punctate/inclusion bodies and MP filamentous aggregates traffic along the cortical ER. **(a–d)** Time-lapse images from individual expression of the p29:eGFP (•) and co-expression of p29-• and MP-• with mCherry(•)-HDEL (ER marker). An arrow marks the position of the p29 and MP structures at each time point. Right panels trace the way of the p29 and MP structures along the cortical ER. **(a)** Unidirectional movement of the p29 inclusion body along the cell cytoplasm. **(b)** Different punctate bodies highlighted by the yellow, blue, and red arrows traffic at different times along the cortical ER. The fusion between two granules is shown in times of 0–90 s by the yellow arrow. **(c)** Go and stop movement (white and red arrows, respectively) of the MP filamentous aggregates is observed along the cortical ER. Fragmentation of the MP aggregates is visualized at time 70–80 s. The frames were each obtained every 10 s and a total of 50 frames. Red and black bars correspond to 10 and 50 μm, respectively.

As previously reported for other plant viruses, classes of viral proteins have the ability to traffic intracellularly (Yang et al., 2005; Harries et al., 2009a,b; Feng et al., 2013, 2016). In this work, during the confocal visualization of the expression of the CiLV-C proteins, a constant motion of the p29 structures (punctate and inclusion bodies), and MP filamentous aggregates throughout the cortical cytoplasm were noticed. The mobility of the p29:eGFP structures was confirmed by time-lapse images showing the position of punctate and inclusion bodies at different times along a row mapping the overall motion (Figure 6a, Movie S1). The same motion pattern was observed by individual expression of the MP:eGFP (Movie S2).

Given the co-localization of p29 with ER/actin and MP with ER, we asked whether or not these cellular organelles play a role in supporting the movement of p29 bodies and MP filaments. The time-lapse observation, firstly from co-expression of p29:eGFP and ER marker (mCherry-HDEL), revealed several p29:eGFP punctate bodies of different sizes, closely associated with ER being directed by the ER network (Movie S3). The movement of individual punctate bodies was tracked, revealing unidirectional movement at different times (Figure 6b, arrows) with the fusion of the structures resulting in larger bodies (inclusions) (Figure 6b; yellow arrow and (Movie S3).

We then examined the role of actin filaments with p29 trafficking. Time-lapse images of cells co-expressing p29:eGFP and actin marker (dsRFP-Talin) revealed an apparent decrease in p29 mobility in the cortical cytoplasm (data not shown), where p29:eGFP punctate structures associated along the actin filaments were extensively observed (as shown in the Figure 5bi). Unfortunately, we could not track the movement; however, this does not exclude that p29:eGFP structures could traffic by microfilaments, given that the complex is continuously distributed along the actin filaments.

In order to verify if the viral MP was carried through the ER network, time-lapse video of plant cell co-expressing MP:eGFP and ER marker was performed. Movement of MP filamentous aggregates along the ER network was observed (Movie S4). In a more detailed view, by time lapse images, decoupling of MP aggregates with resultant motion by ER was observed (Figure 6c, white arrow). However, some MP aggregates associated with ER network remained static for a long observation time (Figure 6c, red arrow).

These findings reaffirm the results of co-localization of the p29 with ER/actin and MP with ER, and show that the putative coat and movement proteins of CiLV-C have the capability to traffic intracellularly by using the ER system.

### Homologous and heterologous interactions between CiLV-C proteins in living plant cell

**Figure 7:**
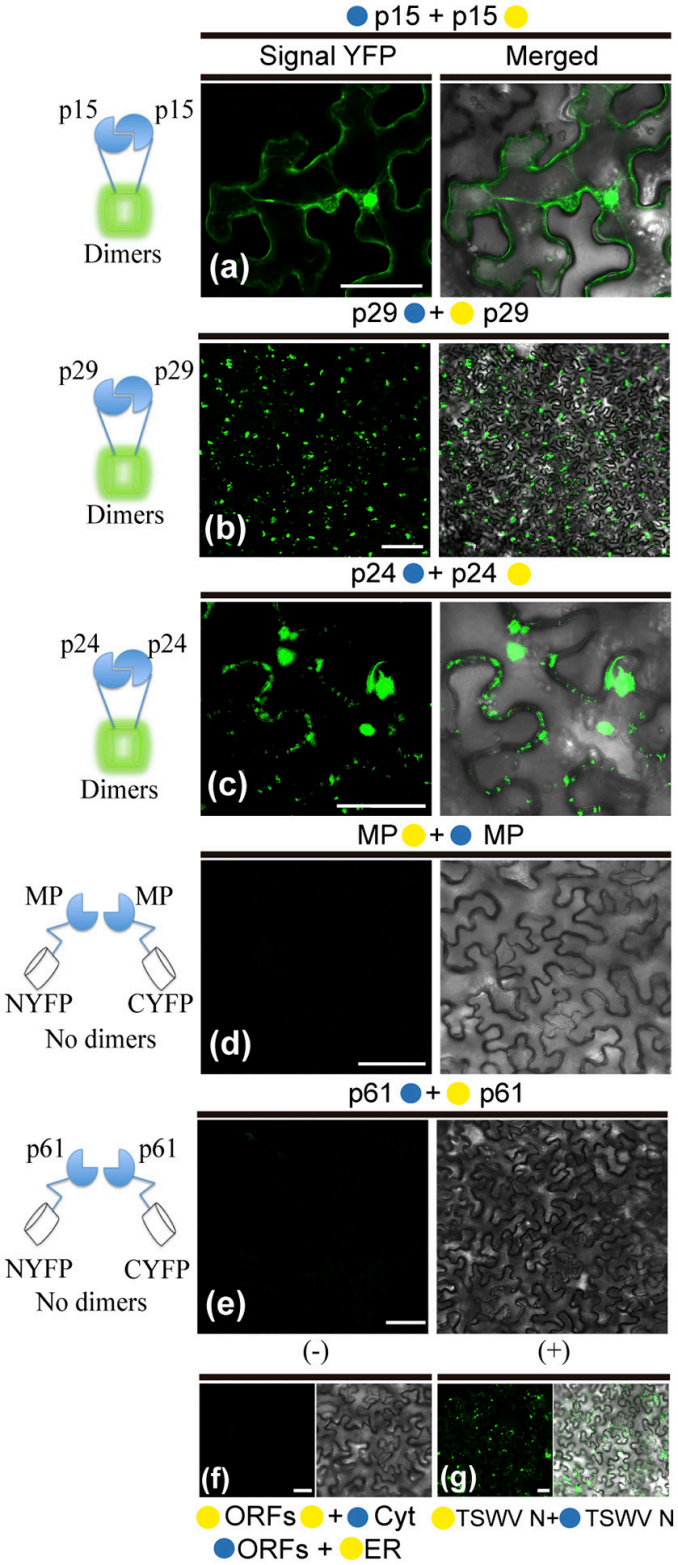
BiFC analyses of dimer formation between proteins of Citrus leprosis virus C. p29, p15, p61, MP, and p24 carrying N-terminal (•) or C-terminal (•) YFP fragments fused at their N- (•/•–ORFs) or C-termini (ORFs-•/•) were transiently co-expressed in *N. benthamiana* leaves by agroinfiltration. Confocal microscopy images corresponding to the representative fusion protein pair combination assayed for p29-p29, p15-p15, p61-p61, MP-MP, and p24-p24 interactions (a synthesis of all protein pair combination performed) are shown in Table 1. All images contain two pictures corresponding to the YFP signal or merged with bright field. **(a)** p15 dimerization with YFP signal located in the nucleus and cytoplasm. **(b)** p29 dimerization with YFP signal located throughout cytoplasm forming inclusions and punctate bodies. **(c)** p24 dimerization with YFP signal in format of vesicle-like spherical structures dispersed by cytoplasm. **(d,e)** No fluorescence return from MP or p61 BiFC expression. Negative controls correspond to the expression of the CiLV-C proteins in combination with Cyt or ER BiFC vectors **(f)**. Positive control corresponds to the expression of the TSWV N protein in BiFC construction, which is known to form dimers *in vivo* **(g)**. The left of each image has a representative scheme of the positive (dimers) or negative (no dimers) interaction corresponding to all proteins assayed. For BiFC, positive interaction means physical association between two putative interaction partners, generating proximity of YFP fragments, thus ensuring the reconstitution of the molecule and the visualization of fluorescent signal by excitation (Kerppola, 2006). Bars correspond to 50 μm.

To further dissect molecular details of the viral proteins, we decided to evaluate by Bimolecular Fluorescence Complementation (BiFC) assay the ability of p29, p15, p61, MP, and p24 to dimerize and to interact with each other *in vivo*. To do this, the N-YFP and C-YFP fragments were fused to the N- and C- terminus of each CiLV-C protein, and transient expression of the different fusion protein pair combinations was performed (Table 1) by agroinfiltration of *N. benthamiana* leaves. For dimer formation, we observed that p15 protein does not have preference for the location of the YFP fragment, resulting in clear reconstitution of the fluorescence with nuclear and cytoplasmic localization in all fusion protein pair combinations evaluated (Table 1, Figure 7a). The p29 punctate and inclusion bodies dispersed by the cytoplasm were visualized for all positive dimer combinations of the p29 protein (Figure 7b) with restriction in the reconstitution of the fluorescence only for the pair combination N-YFP:p29 + C-YFP:p29 (Table 1), which had its N-terminus fused. The opposite was observed for p24 protein with fluorescence visualized only in the pair combination p24:N-YFP + p24:C-YFP that had its N-terminal non-fused to the YFP fragment (Table 1). YFP signal in a spherical format was revealed from p24 dimerization (Figure 7c). The dimerization of the p15, p29, and p24 proteins reproduced the same fluorescent pattern obtained with the respective proteins fused at their C-termini with the GFP, further suggesting that this region of fusion does not alter the intracellular localization of the proteins. Intriguingly, in all MP fusion pair combinations, we did not visualize fluorescence (Table 1, Figure 7d). In order to exclude the possibility of false negatives, we performed the co-expression of the combination MP:N-YFP + C-YFPcyt, which resulted in clear YFP signal in punctate structures along the cell periphery (see findings in topology assays, Figure 9Fxviii). Also, we did not visualize the signal from any of the p61 fusion pair combinations (Figure 7e); probably due to the activation of necrosis in a short period of time after p61 infiltration into the plant leaves (as aforementioned). In this respect, BiFC methodology was inconclusive to state that p61 protein does not dimerize *in vivo*. Due to this fact, we decided to remove this protein of the heterodimerization assay. No fluorescence signal was observed when the p15, p29, and p24 had their N-or C-terminus fused to the C-YFP fragment and were co-expressed with counterpart N-YFP target to the cytosol, and when their N-terminus fused to the N-YFP were co-expressed with counterpart (C-YFP) target to the ER, (negative controls, Figure 7f). We used the nucleocapsid (N) of tomato spotted wilt virus (TSWV) as positive control, given its ability to form dimers, as previously reported (Uhrig et al., 1999; Leastro et al., 2015). As expected, clear fluorescence signal was visualized by co-expression of the combination C-YFP:TSWV N + N-YFP:TSWV N (Figure 7g).

In general, these results show the dimerization of the p29, p15, and p24, and absence of MP self-interaction *in vivo*. They also indicate that p15 does not distinguish its N- or C-terminal for interaction, while the p24 N-terminal region seems to be essential for dimerization, and the p29 form dimers either in their N-N termini or in the C-N termini interface.

**Figure 8:**
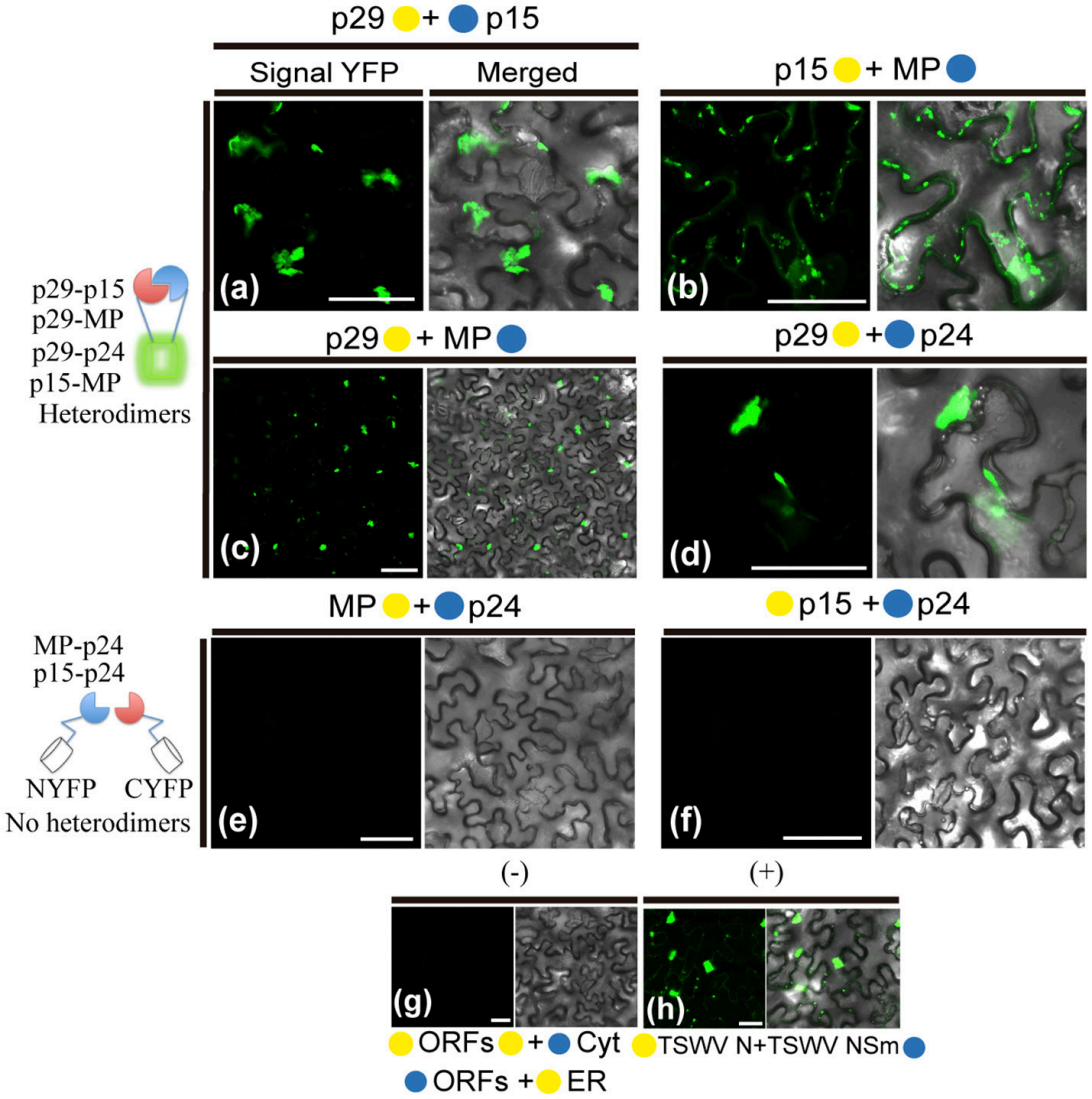
BiFC analyses of heterodimerization between proteins of Citrus leprosis virus C. p29, p15, MP, and p24 carrying N-terminal (•) or C-terminal (•) YFP fragments fused at their N- (•/•–ORFs) or C-termini (ORFs-•/•) were transiently co-expressed in *N. benthamiana* leaves by agroinfiltration. Representative protein pair combinations are indicated at the top of each image; all hetero-combinations performed are shown in Table 2. **(a–d)** Correspond to positive interactions (heterodimers: p29-p15, p29-MP, p29-p24, and p15-MP), summarized in the scheme to the left of the images. **(e,f)** Correspond to non-interaction (no heterodimers: MP-p24 and p15-p24) also summarized in the scheme. Negative controls correspond to the expression of the CiLV-C proteins in combination with Cyt or ER BiFC vectors **(g)**. Positive control corresponds to the expression of the N and NSm proteins of the TSWV in BiFC construction, which is known to interact *in vivo* **(h)**. Bars correspond to 50 μm.

The hetero-association between proteins was also evaluated. Reconstitution of the fluorescence was observed for the combinations between p29-p15, p15-MP, p29-MP, and p29-p24 (Figures 8a–d, respectively). On the other hand, the co-expression between MP-p24 and p15-p24 did not generate fluorescence (Figures 8e,f, respectively). The same negative controls performed in the dimerization assay also it applies to heterologous interaction (negative controls, Figure 8g). In this assay, we used as positive control the cell-to-cell movement (NSm) and N proteins of TSWV, which have been shown to interact from different methods (Soellick et al., 2000; Leastro et al., 2015). As expected, the expression of the protein pair combination C-YFP:TSWV N + TSWV NSm:N-YFP generated the fluorescence signal (Figure 8h). Table 2 summarizes all findings of the transient co-expression, and reports all different fusion protein pair combinations performed. The images displayed are representative of at least three independent experiments. In all cases, the expression of the corresponding fusion proteins was confirmed by Western blot analyses, using specific antibodies that recognize the N- or C-terminus of the YFP protein (data not shown).

### CiLV-C p29, p15, MP, and p24 are membrane-bound proteins

Hydrophobic prediction analyses from CiLV-C proteins were performed with various transmembrane prediction tools to determine the regions of the proteins that might enable interaction with membranes. The prediction results varied according to the parameters used. TMHMM server (http://www.cbs.dtu.dk/services/TMHMM/), Phorbius (http://phobius.sbc.su.se) and MPEx (Snider et al., 2009) predicted that p29, p15, and MP did not have transmembrane domains (TMD). On the other hand, the p61 was predicted to have one, three or five TMDs, respectively, and p24 four TMDs (Figure S4A). Although no membrane-spanning domains were identified for MP and p29, the MPEx hydropathy analyses predicted one hydrophobic region (HR) for p29 that encompassed residues 176-194 and three for MP: HR1 48–66, HR2 88–106 and HR3 173-191 (Figure S4B). For p24 three hydrophobic regions were predicted: HR1 42–60, HR2 86–104, and HR3 114–172. The latter encompassed 58 residues that correspond to two potential TMD (Figures S4A,B). In addition, coiled coil regions were predicted for p29 and p24 (COILS Prediction Server) (Figure S4C). Coiled coil viral protein structures are known to interact with lipid bilayer transforming the ER membrane to vesicle, and allowing interaction among viral proteins (Stavolone et al., 2005; Leastro et al., 2015; Singh and Savithri, 2015). Taken together, these prediction analyses suggest that p24, p61, MP, and p29 are potentially membrane-associated proteins.

**Figure 9:**
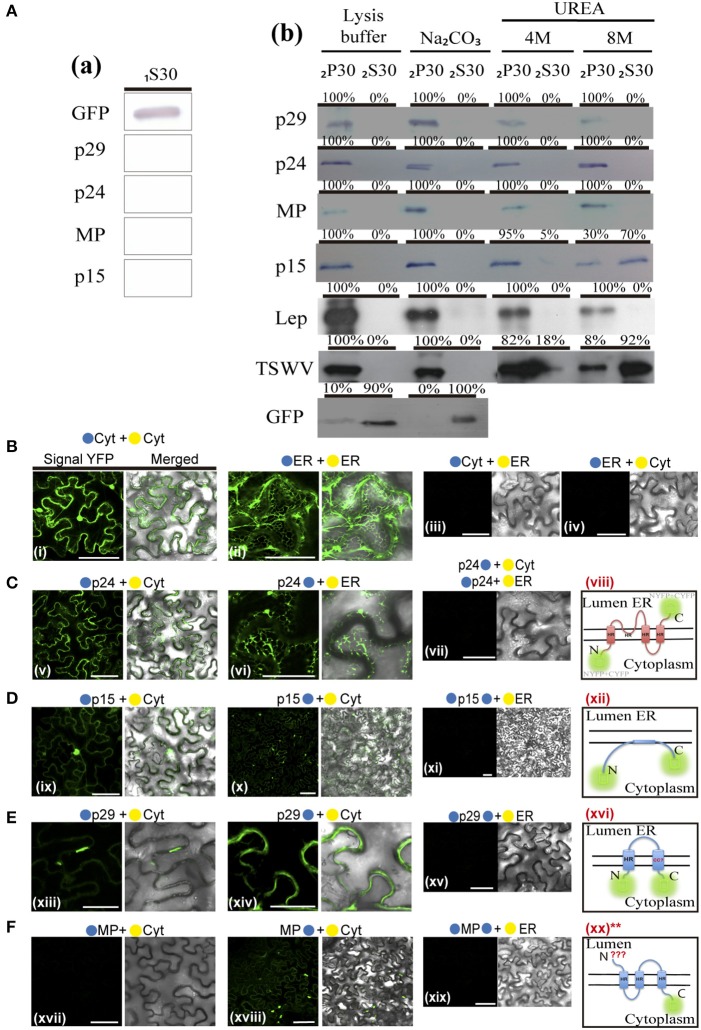
Membrane association and topology of citrus leprosis virus C proteins. **(A)** Segregation into membranous and soluble fraction of p29, p15, MP, and p24 CiLV-C proteins expressed *in planta*. Respective proteins targeted with eGFP were expressed in *N. benthamiana* leaves by agroinfiltration. As control, we used leaf protein extracts containing unfused expressed eGFP, the HA-tagged LEP (leader peptidase) protein and HA-tagged NSm protein of TSWV, which have been showed to be non-membrane, integral membrane, and peripheral membrane proteins, respectively. The supernatant from first ultracentrifugation before membrane fraction (_1_S30) **(a)**, and comparable pellet (_2_P30) and supernatant (_2_S30) obtained after membrane fractioning, untreated and alkaline or urea (4 or 8M) treatments **(b)**, were analyzed by Western blot method using an anti-NtGFP antibody (Sigma^TM^) or anti-HA antibody (Fermentas^TM^). Relative quantification values are presented in percentage. **(B-F)** Subcellular localization (cytosolic face or ER lumen) of the N- or C-termini of the p24 **(C)**, p15 **(D)**, p29 **(E)**, and MP **(F)** proteins. All proteins carrying the N-terminal (•) or C-terminal (•) YFP fragments fused at their N-(•/•–ORFs) or C-termini (ORFs-•/•) were transiently co-expressed in *N. benthamiana* leaves with corresponding complementary YFP fragment addressed to the cytosol face (•-cyt or •-cyt) or the lumen of the ER (•-ER or •-ER). Images reveal the topology of the N-terminus (v, ix, xiii, and xvii) or C-terminus (vi, x, xiv, and xviii) of the respective CiLV-C proteins. Positive and negative controls are presented in pictures **(B)**. Hypothetical topologic models are presented at the panels to the right of the figure for each respective protein (viii, xii, xvi, and xx). HR = hydrophobic region, N = N-terminus, C = C-terminus, CC? = possible membrane span coiled coil structure. **Correspond to the hypothetical model for MP, where only the localization of the C-terminus of the protein was characterized. All images contain two pictures corresponding to the YFP signal or merged with bright field. The fluorescence was monitored 4 days post-infiltration using a confocal Leica microscope SP8 model. Bars correspond to 50 μm.

Based on these predictions and in order to examine membrane association of CiLV-C proteins, we prepared subcellular microsomal fraction from agroinfiltrated *N. benthamiana* leaves transiently expressing the p29, p15, MP, and p24 proteins fused to the eGFP in their C-termini. As control, we used leaf protein extracts containing unfused expressed eGFP, the HA-tagged LEP (leader peptidase) protein, and HA-tagged NSm protein of TSWV, which have been shown to be non-membrane, integral membrane, and peripheral membrane proteins, respectively (Peiro et al., 2014; Kang et al., 2015; Leastro et al., 2015). The p61 protein was removed from the assay due to its apparent hypersensitive response, which hinders the protein expression analysis. p15 was also assessed, despite the fact that this protein does not present, from prediction, HR or TM domains. Plant tissues expressing the respective proteins above mentioned were lysed, and extracts were separated by high-speed ultracentrifugation into pellet (P30) and supernatant (_1_S30) fractions. Immunoblot analysis showed the marking of the soluble fraction (_1_S30) containing free eGFP only (Figure 9Aa). The P30 fraction was resuspended and subjected to the chemical treatments with lysis buffer, Na_2_CO_3_ (pH 11.5), 4 and 8M urea. To identify the type of interaction, we first washed the membrane-rich fraction from each sample with Na_2_CO_3_, a treatment that is known to render microsomes into membrane sheets, releasing soluble luminal proteins (Peremyslov et al., 2004). The results showed that all tested CiLV-C proteins remained associated with the membranous fraction (Figure 9Ab; _2_P30 100% for p29, p15, MP, and p24), suggesting that CiLV-C proteins are tightly associated with membrane. As expected, the proteins control Lep and NSm also remained associated with the membranous fraction (Figure 9Ab, _2_P30 100% for Lep and NSm). In contrast, the free GFP protein control remained in the soluble fraction (Figure 9Ab; _2_S30 100%). After 4M and 8M urea treatment - a treatment that should release all polypeptides bound to the membrane, except for the integral membrane proteins (Martínez-Gil et al., 2009; Leastro et al., 2015) - the eGFP-tagged p29, MP, and p24 proteins (in both 4 and 8M) remained in the pellet fraction (_2_P30 100% for p29, MP, and p24, Figure 9Ab). The same accumulation was observed for Lep protein (Figure 9Ab), indicating that these CiLV-C proteins are fully integrated to membranes. In contrast, after a more aggressive treatment (8M urea), the majority of the eGFP-tagged p15 protein (about 70 %), was associated to the soluble fraction (Figure 9Ab), such as observed for the NSm protein (_2_S30 92%). This suggests that p15 might be a peripheral protein tightly associated to membrane, although the membrane interaction region, curiously not predicted, probably does not span the lipid bilayer.

Given the association of the CiLV-C proteins with membrane, the BiFC assays were performed in order to determine the subcellular compartments in which the N- or C-terminus of the membrane proteins are exposed. The same BiFC constructs containing the CiLV-C ORFs fused at their amino- or carboxy-termini, the N-YFP or C-YFP fragments used in the dimer/heterodimer assay, here were transiently co-expressed with the counterpart of EYFP target to the cytosol (C-YFPcyt or N-YFPcyt) or the ER (C-YFPer or N-YFPer). All protein pair combinations are shown in Table 3. Reconstitution of the fluorescence-competent EYFP structure indicated the *in vivo* localization of the fused/inserted YFP half in the appropriated compartment. Confocal microscopy images revealed no fluorescence signal when the two EYFP were co-expressed in different subcellular compartments (N-YFPcyt + C-YFPer or N-YFPer + C-YFPcyt; Figures 9Biii,iv). However, fluorescence reconstitution was observed when EYFP fragments were co-expressed in the ER or cytosol (N-YFPcyt + C-YFPcyt or N-YFPer + C-YFPer; Figures 9Bi,ii). Next, we co-expressed the p29, p15, MP, and p24 CiLV-C proteins attached to their N- or C-termini, the N-YFP or C-YFP fragment with the counterpart of EYFP target to the cytosol or the ER. For p24, the YFP fluorescence signal was visualized only in the co-expression of the pair combinations p24:N-YFP + C-YFPer and N-YFP:p24 + C-YFPcyt (Figure 9C), indicating that in ER plant cells, the N-terminal region of p24 is exposed to the cytoplasm whereas the C-terminal domain faces the ER luminal side. For the p15 and p29, reconstitution of the fluorescence was only observed when the respective proteins, carrying the N-YFP fused to the N-or C-terminal, were co-expressed with C-YFPcyt, indicating that p15 and p29 were exposed to cytosolic compartment (Figures 9D,E, respectively). For MP, curiously we visualized reconstitution of fluorescence only of its C-terminal exposed to cytosolic compartment, combination MP:N-YFP + C-YFPcyt (Figure 9F).

**Table 3:** Synthesis of all protein pair combinations performed in the BiFC topology assay for p29, p15, MP, and p24 CiLV-C proteins.

**Membrane topology**	**NYFP-Cyt**	**CYFP-Cyt**	**NYFP-ER**	**CYFP-ER**
NtYFP-ER		–		+++
NtYFP-Cyt		+++		–
p29-NtYFP		+++		–
p29-CtYFP	–		–	
NtYFP-p29		+++		–
CtYFP-p29	–		–	
p15-NtYFP		+++		–
p15-CtYFP	–		–	
NtYFP-p15		+++		–
CtYFP-p15	–		–	
MP-NtYFP		+++		–
MP-CtYFP	–		–	
NtYFP-MP		–		–
CtYFP-MP	–		–	
p24-NtYFP		–		+++
p24-CtYFP	–		–	
NtYFP-p24		+++		–
CtYFP-p24	–		–	

*The symbols –, +, ++, and +++ correspond to the absence, low, medium or high fluorescence signals, respectively. NYFP and CYFP correspond to half N- and C-terminal of the YFP protein, respectively*.

## Discussion

### CiLV-C infection, subcellular localization, intracellular trafficking and homologous and heterologous interactions of the proteins

It has been suggested that CiLV-C accumulates and replicates in the cytoplasm of infected host cells (Knorr, 1968; Kitajima et al., 1974; Bastianel et al., 2010). Our studies on intracellular localization, traffic, and interactions of CiLV-C proteins further enhance this evidence. Here, confocal images exhibited ER remodeling by interaction with p24, resulting in the formation of vesicle-like spherical structures ranging in size from 800 nm to 10 μm (predominantly ca. 1.5 μm). In bromoviruses, the sole expression of the non-structural 1a protein was also sufficient to ensure membrane rearrangements in the ER lumen, given rise to vesicle spherules that correspond to the sites of viral RNA replication (Schwartz et al., 2002). Just as happened from expression of the p24, the ER disorders were seen from p15 association with ER. Curiously, on the context of CiLV-C infection, ER membrane deformations were extensively observed, with the formation of pleomorphic, mostly circular, vesicle-like structures, encompassing clusters of short bacilliform virions (Figures 1B,C), which strongly suggest places on ER for virus replication and assembly. These structures resemble those observed from individual expression of the p24 and p15, suggesting that these proteins may be responsible for their formation. These data allow us to hypothesize that p15 and p24 proteins could have a possible role with the formation of viral replication complexes in the infection process of CiLV-C. Similar membrane changes and vesicular structures are extensively observed, in infection processes, for most families of RNA viruses, often resulting in the formation of specialized structures (VRCs) involved in the protection of the machinery for replication and virion assembly (Schwartz et al., 2004; Adams and Antoniw, 2005; Mandahar, 2006; Laliberte and Moss, 2010; Laliberté and Sanfaçon, 2010; Genovés et al., 2011).

Furthermore, the p24 is homolog of virion membrane proteins of plant and insect viruses (SP24 family protein), and are thought to be the main viral membrane protein (Kuchibhatla et al., 2014). Considering this predicted structural aspect (confirmed in this present work from microsomal fraction and topological assays), its capability in inducing membrane deformation and the fact that CiLV-C has an enveloped bacilliform particle, the set of these data strongly suggest that p24 might have a structural role, as the viral matrix protein. This aspect further strengthen our hypothesis about the role of the protein in the formation of replication sites, considering that matrix proteins play critical role in virion morphogenesis by assembly and budding, and also promote the release of virus-like particle (VLPs) resembling the immature virion (Neumann et al., 2009; Battisti et al., 2012). Although speculative, it is tempting to hypothesize that the p24 might be of such importance for CiLV-C infection and could also be structurally involved with stability of the viral particle.

In addition to the ER, p15 was also found in the nucleus; even though neither nuclear localization signals (NLS) nor nuclear exportation signals (NES) were detected (Table S1). We believe that its transport to the nucleus may occur through passive diffusion, given the small size of the complex (p15 plus GFP = 42 kDa). Nuclear pore complexes allow passive diffusion of small proteins and even greater than 60 kDa (Wang and Brattain, 2007). Furthermore, heterodimerization assay reinforces our hypothesis, once after heterologous association of the p15 with p29 and MP there was no routing of the protein complex to the nucleus (Figures 7a,b), suggesting the lack of p15 functionality in this organelle. To date, there are no records of nucleus involvement in CiLV-C infection process, in contrast to what is extensively observed in the infection of dichorhaviruses also transmitted by *Brevipalpus* mites to citrus, such as orchid fleck virus strain citrus and citrus leprosis virus N (Bastianel et al., 2010; Ramos-González et al., 2017). We did not find any inferences about the structural role of the p15 in virion composition, and hence its involvement may relate to the infection process. Recently, it was suggested a putative Zn-finger motif located in the C-terminus of the p15 protein (Ramos-González et al., 2016). This inference, together with the capability of the protein to remodel the ER, allows us to speculate the potential for recognition of the viral genome by the protein within this compartment, which could be involved with replication of the virus genome.

Our prediction analysis from residues of p61 shows the presence of a signal peptide in its N-terminal (cleavage site between position 15 and 16), followed by four N-glycosylation sites along the protein, and the presence of 3 TMDs in its extreme C-terminal (Table S1, Figure S4A). Thus, p61 has all features of a virion glycoprotein. In addition, a combination of powerful automated and in-depth manual analysis identified homology of putative glycoproteins ORF2 of insect viruses from taxa *Chroparavirus* and Negevirus, with the cilevirus p61 showing similar features among them that indicate possible equivalent function as glycoproteins (Kuchibhatla et al., 2014). Our confocal studies demonstrate that p61 remodels the ER and co-localizes with the Golgi apparatus. Similar localization was reported when the precursors (Gn and Gc) of the glycoproteins (Gp) of a tospovirus were co-expressed with ER and Golgi marker in plant cells. The proteins were able to associate with ER and GC and to induce membrane deformation, with the formation of pleomorphic vesicle-like structures (Ribeiro et al., 2008). Herein, the same pattern was observed from association of p61 end ER, resulting in ER remodeling with the formation of globular structures along the ER network (possibly vesicle-like structures); however, the same membrane deformation was not observed from association with GC. Surprisingly, a distinct phenotype of cytoplasmic redistribution of Golgi apparatus has been observed here, where it is clearly possible to evaluate a consistent co-localization, suggesting a strong interaction of p61 and Golgi, which may be involved with viral protein processing. Nevertheless, p61 has the capability to induce membrane deformation, a phenomenon that may be involved in the formation of viral lipid envelope, such as proposed for other membrane enveloped viruses (Latham and Galarza, 2001; Shaw et al., 2003; Kolesnikova et al., 2004; Ribeiro, 2008). Given strong evidences of glycoprotein function for p61, we hypothesize, such as for p24, its potential involvement in the particle assembly, which could also give rise to the formation of VLPs, since we observed that this protein is also able to induce membrane deformation in the ER. However, whether or not ER remolding originated from p61 and p24 expression has biological sense in the VLP formation requires additional investigation.

The images of co-expression between the p61:eGFP construction and subcellular marks (co-localization assays) were obtained with great difficulty, performed several times in 24 or 36 h post-infiltration, always seeking for regions of the tissue not yet affected by necrosis. The visualization of fluorescence by BiFC assay requires viable fusion proteins being expressed for a long time in the cell in order to favor the chemical reaction required for formation of the cyclic fluorophore (Kerppola, 2006). Unfortunately, it was not possible to analyze by BiFC the *in vivo* association of p61 (dimerization), and interaction with other CiLV-C proteins (heterodimerization) due to the fast necrotic response on the infiltrated leaves, an additional data that could reinforce our interpretation about the functionality of this protein. This necrotic response may also justify the low number of fluorescent cells from the p61 expression fused to the eGFP, and the non-labeling of the protein in Western blot analyses. To better understand this response, an interesting analysis would be to evaluate the possible association of the p61 protein with cell receptors involved with the machinery that activates or regulates cell death in plants.

Studies on the extensively investigated tobacco mosaic virus (TMV) have demonstrated that cytoskeletal microfilaments and associated motor proteins play important role in the intracellular movement of viral replication complex, whereas ER membranes that traffic VRCs and MPs are essential in the cell-to-cell movement of the MP and virions between cells (Liu et al., 2005; Guenoune-Gelbart et al., 2008; Harries et al., 2009b). Our data demonstrate that p29 (putative coat protein) is associated with ER and actin, and may traffic utilizing the ER network. The same feature was recently observed for the N protein of TSWV, assigning a novel property to capsid proteins of plant viruses on intracellular trafficking (Feng et al., 2013). Considering that viral capsid proteins have essential role in viral replication and transcription (Bol, 2008), there is a possibility that intracellular trafficking of the p29 has involvement with the transport of the CiLV-C replication complex or vRNP to the cell periphery, thus facilitating the passage of the viral RNA to neighboring cells. Curiously, we observed the interaction between p29 and MP and found the MP occurs in cell periphery associated with PD. In addition, we identified that the CiLV-C MP not only interacts with the ER but also travels by the ER network, similarly to what we observed for p29. Although we did not see the mobility of p29 structures along the actin, due to overexpression of dsRFP-Talin that partially or completely inhibit the myosin-dependent movement by disturbing the actin-myosin interplay, such as previously reported (Holweg, 2007; Genovés et al., 2010), it is likely that the p29 protein may be capable of moving in an actin-dependent intracellular flux. This, given our results of positive co-localization of p29 bodies along the actin filaments, suggests a possible routing by the microfilament. Corroborating our data, similar co-alignment pattern was observed in other studies (Holweg, 2007; Harries et al., 2009b; Genovés et al., 2010; Feng et al., 2013), helping understand the intracellular mobility of viral proteins through the actin system.

All these findings suggest that MP and p29 may work together to ensure the viral spread into the cell and to neighboring cells from the use of the actin/ER system as a route to move the viral components (VRCs and/or RNPs) cell-to-cell. Viral movement proteins assure the cell-to-cell transport via association with the cell wall connections known as PD (Lucas, 2006; Fernandez-Calvino et al., 2011); in some cases both MP and CP are required for cell-to-cell and systemic movements (Scholthof, 2005; Lucas, 2006). Whether or not the spread of the virus is actin- or myosin-dependent, and the direct effect of these proteins and organelles on the viral movement still needs to be addressed. Unfortunately, the lack of a reverse genetics system for CiLV-C precludes a more detailed study about some of these aspects, principally given the difficulty of viral mechanical inoculation, inoculation via vector (given its arduous work of *Brevipalpus* mites manipulation), and the natural limitation of the virus in ensuring systemic infection in the plant.

CiLV-C MP has basically all features that fits as a viral movement protein: it is a homolog of the 30K superfamily of viral movement protein; it has association with PD, suggesting activity in the cell movement, and traffics by the endoplasmic reticulum system; however, it has some unique features. The p30 movement protein of TMV is known to form dimers, and it is suggested that the oligomerization of MP may play a role in the aggregation of ER-containing viral replication complex (Brill et al., 2004). Such oligomerization is also important for viral pathogenesis in members of genus *Potexvirus*, where mutation in sorting signals of TGBp3, essential for protein dimerization, impairs viral spread (Wu et al., 2011). Here, from *in vivo* BiFC assay, the CiLV-C movement protein did not self-interact. Considering that CiLV-C has a natural limitation in ensuring the systemic viral spread, though speculative, there is a possibility that the inability of the MP to interact *in vivo* may have direct involvement with this phenotype. Experiments that evaluate the efficiency of viral movement from the insertion of the CiLV-C MP in heterologous system, i.e., TMV (Lewandowski and Adkins, 2005) or *Alfalfa mosaic virus* (AMV) model systems (Sanchez-Navarro et al., 2001; Leastro et al., 2017) will help to clarify this question. It is important to note that some protein interactions may only occur in the presence of virus replication, requiring more than one interaction partner (Dietzgen et al., 2012). In this sense, it will be important to examine whether or not the CiLV-C MP has the capability to self-interact from transient expression in infected plant. Conversely, to evaluate the dimerization by alternative protein interaction assay, such as pull-down or yeast-two hybrid system, would help to clarify this question. Collectively, these additional tests would better assist in understanding the movement machinery used by the virus.

Part of the cytopathology observed here from individual expression of CiLV-C proteins has already been viewed for other viral species (i.e., ER changes). However, we identified new details, on the process through which viral proteins interact with cell organelles and modify them. In addition to what has been reported so far in this study, we further observed association of the p24 and MP with GC and p24 with actin filaments. On the context of infection, as mentioned above, the p29 and MP interact and may work together to ensure viral spread. Given that p24 interacts with actin and associates solely with p29 among all CiLV-C proteins studied, there is possibility that p24 may also participate in viral spread within the cell. We did not find any biological sense on the involvement between p24 and MP with Golgi; however, these associations may indicate host interaction or additional function to these proteins. Thus, in the future, we intend to study such a process in more detail, in order to correlate the cytopathology observed here with possible biological functions in viral infection. We do not rule out a possible artificial effect by overexpressing CiLV-C proteins under the control of CaMV 35S promoter. However, all these structures shown in co-localization were consistently present in many cells in different transformed plants exhibiting patterns different from those observed by the sole expression of GFP or its co-expression with organelle markers.

Homologous and heterologous interactions are known to play significant role in the functioning of different proteins. An interesting observation is that the p29 forms dimers both with its N-C and N-N termini free interfaces (not attached with YFP halves), which may indicate interactions both in the head-to-tail (N-C terminal) and in the head-to-head (N-N terminal) interfaces. This observation suggests a plasticity in the p29 dimerization, which may facilitate conformational changes of the virion structure during morphogenesis. This finding corroborates the broad permissibility of interacting, *in vivo*, evaluated by BiFC method for nucleocapsid proteins of different tospoviruses, where similar plasticity was reported; however, in all configurations N-C, N-N, and C-C (tail-to-tail) (Leastro et al., 2015). The set of findings presented here for p29 (intracellular localization, cytoplasmic trafficking, and dimerization), with previous reports of abundant p29 subgenomic expression (Pascon et al., 2006) and *in situ* labeling of virions and the viroplasm with specific p29 antibody (Calegario et al., 2013; Garita et al., 2014), strongly supports the idea that p29 is the capsid protein of the citrus leprosis virus C (CiLV-C CP).

During confocal visualization of the NYFP:p24 and GFP:p24 expression with its N-terminal attached, we observed absence of vesicle formation; curiously, p24 formed dimers only in a C-C terminal interface. These findings suggest that its extreme N-terminal may be essential to interact with cell membrane. Furthermore, *in silico* analysis predicted HR region and a coiled-coil domain at the N-terminal, representing a potential region to interact with membranes for vesicles formation (Leastro et al., 2015; Singh and Savithri, 2015).

The p15, similarly to p29, showed high plasticity in the dimerization, but in this case without any restriction, suggesting associations at all interfaces of the molecules (N-N, C-C, and N-C), probably due to a command tertiary structure. These observations support the idea that compatibility of interaction among different regions of a protein with itself and among other proteins must be essential for the development of infection.

The tentative biological meaning of the positive interactions among different proteins (hetero-association) will be explored in the last topic of this discussion (hypothetical model).

### Membrane association and topology

Plant viruses induce substantial cellular remodeling during infection. In particular, the association of viral proteins with membranes has important role for successful infection, mainly ensuring the formation of sites (vesicles) for viral replication and virion assembly (Schwartz et al., 2004; Adams and Antoniw, 2005; Laliberté and Sanfaçon, 2010; Lorizate and Kräusslich, 2011).

The microsomal fraction assays revealed that basically all CiLV-C proteins assessed are associated with membranes. The membrane associated-pattern reported here for the CiLV-C proteins is aggregated with the pattern reported for different membrane proteins of other plant viruses (Peremyslov et al., 2004; Genovés et al., 2011; Kang et al., 2015). These findings further support the association of the viral proteins with ER network and Golgi complex, and possibly this association occurs through a direct interaction between the proteins with the membranes of the organelles.

The BiFC analysis revealed that the p24 N-terminus is exposed to the cytoplasmic face, whereas its C-terminus is located at the ER lumen. However, prediction analysis showed four potential TMD for this protein (Figure S4A), which does not correspond to the characterized topology. Based on TMD prediction, in confocal analyzes we expected to observe that both extremes of the protein should be facing the same cell compartment. However, we conclude the opposite of what was predicted. These results suggest that one of the four putative transmembrane (TM) segments is membrane associated and corroborate the study performed by (Kuchibhatla et al., 2014). The authors infer that potentially the second putative TM segment could not span the membrane, which is less hydrophobic (ΔG = −1.33) than TM 1 (ΔG = −1.18), TM 3 (ΔG = −0.52), and TM 4 (ΔG = 1.03) (Figure S4B). Future experiments evaluating the membrane association of each TMD will help to conclude this question.

In the structural context of virus particle, it has been suggested (Kuchibhatla et al., 2014) that SP24 family of virion membrane proteins of insect and plant viruses presents a N-terminus containing mostly basic residues, and a C-terminus positively charged (rich in R/K residues) that contains potential N-glycosylation sites (in the case of proteins homologs to p24). This suggests that the N-terminus of the protein is within the virion, which would allow it to bind the viral RNA, while the C-terminus is on the outside of the virion. Interestingly, the topology presented here for p24 protein meets this hypothesis and further supports the idea of the structural role of p24, possibly as a matrix protein (as aforementioned).

Although only one HR is predicted for p29, its integral membrane-associated topology with both N- and C-termini oriented to the cytosolic face suggest the presence of two membrane-spanning domains. Curiously, a coiled coil structure was identified near the HR region on the C-terminus of the protein (Figure S4C); thus we speculate that both structures could be associated with membranes, ensuring this topology. Structural coiled coil motifs have also been implicated in enabling the fusion of the protein with cell membranes both in animal and plant cells (Perraki et al., 2012; Singh and Savithri, 2015). The peripheral membrane topology with both N- and C-termini oriented to the cytoplasm for p15 is extensively observed in many viral proteins involved in different stages of the viral infection cycle (Spaete et al., 1994; Britt and Mach, 1996; Kolesnikova et al., 2002; Earp et al., 2005; Peiro et al., 2014; Leastro et al., 2015).

Intriguingly, the MP did not expose its N-terminus to any of the evaluated subcellular compartment. This point can be explained in two ways: (i) the N-terminal of the MP was not accessible for interaction due to the presence of the attached YFP fragment or (ii) the protein has a C-cytoplasmic and N-luminal topology; however, the ER lumen does not correspond to the compartment that the C-terminal of the protein is exposed, but possibly the lumen of another cell organelle. This hypothesis is based on the prediction of three HR (Figure S4B), in addition to its capability to integrally bind the membrane. All these points suggest that the MP could be a multi-pass membrane protein exposing C-cytoplasm and N-lumen orientation. On the other hand, if the first possibility is correct, it may be a consequence of the inability of dimerization of the MP by BiFC, where the presence of a tag attachment would prevent the correct three-dimensional arrangement of the protein for interaction. However, it is a rather dubious hypothesis, in view of the competence of the protein in hetero-associating, having its N-like C-terminal attached (see Table 2, i.e., heterodimerization between MP and p29). Further experiments of membrane fractionation from each HR of the MP could better clarify the complete topology of the MP.

Altogether, the *in silico* prediction, subcellular fractionation, topological results, and interaction of the proteins with ER allow us to propose the topological model of association of the p24, p15, MP, and p29 with membrane. We infer that p24 is a multi-pass membrane protein with putative transmembrane domains exposing an N-cytoplasm-C-lumen topology (Figure 9Cviii). The p15 is peripherally associated to membrane with the full-length molecule oriented toward cytoplasmic face of the biological membrane (Figure 9Dxii); the same orientation was characterized for p29, nevertheless, it is integrally associated to membrane (Figure 9Exvi). Finally, the MP has its C-terminus exposed to the cytoplasmic face (Figure 9Fxx); however, to uncover the subcellular compartment where the N-terminus is exposed to, further testing is required, but we speculate that it is in the luminal fraction.

### Hypothetical model of replication, particle assembly, and intra- and intercellular spread of citrus leprosis virus C

**Figure 10:**
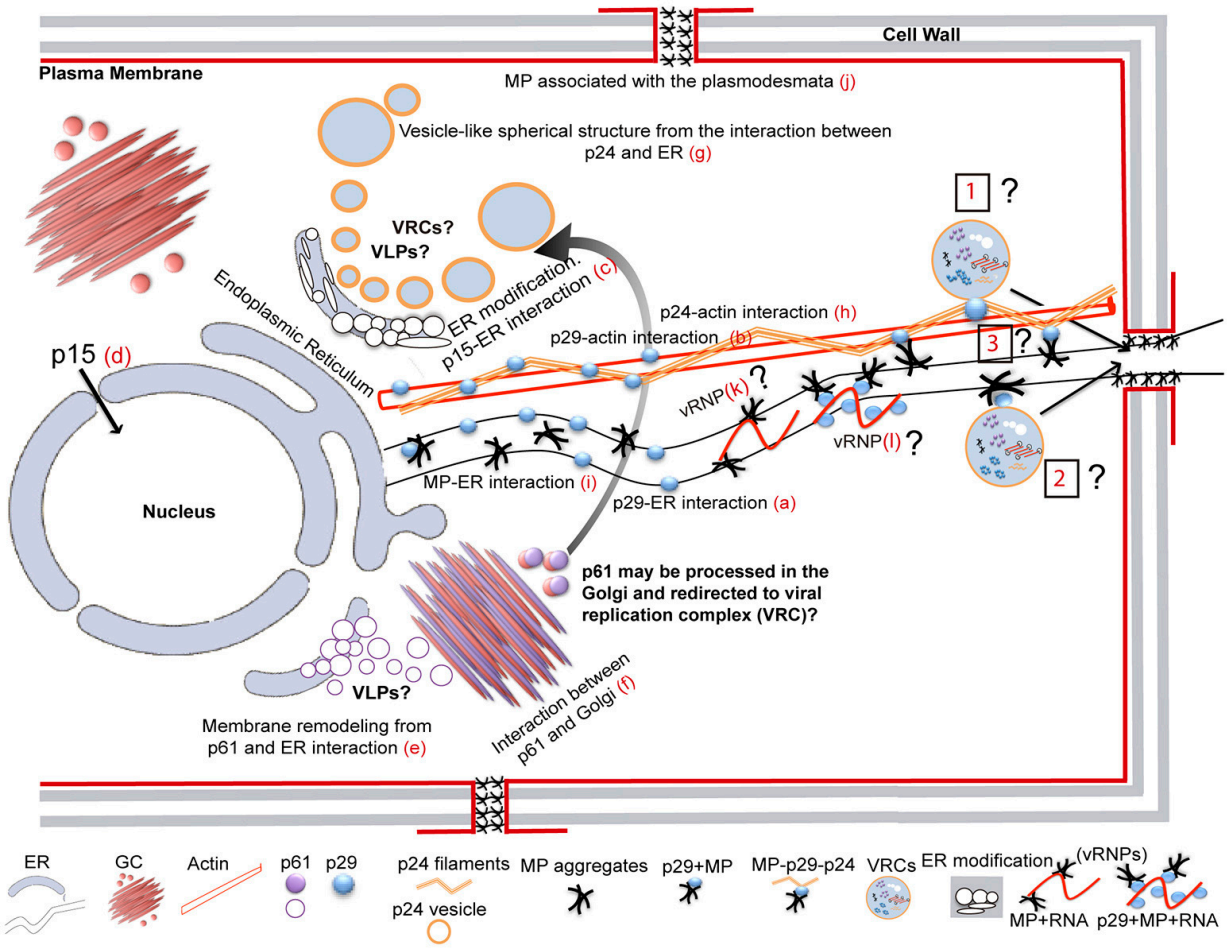
Hypothetical model of replication, assembly and intra- and intercellular spread of citrus leprosis virus C. p29 punctate bodies are associated with cortical ER structure and actin network **(a,b)**. Interaction among p29 with MP and p24 are shown in these organelles. ER modification with pleomorphic formats is formed by the interaction between p15 and ER **(c)**. This protein is also visualized into the nucleus **(d)**. The p61 co-localizes with ER and GC, remodels the cortical ER and forms cytoplasmic aggregates from interaction with GC **(e,f)**. Vesicles-like spherical structures are formed from the interaction between p24 and ER, with the protein surrounding the ER **(g)**. Filament-like structures formed by p24 are wrapped around the actin filaments **(h)**. The movement protein associates with ER network **(i)**, and co-localizes with the plasmodesmata at the cell periphery **(j)**. In addition, curious cytopathic effects are observed by the interaction of p24 and MP proteins with Golgi complex (not shown in this scheme; see **Figure 3**). Based on our findings of CiLV-C proteins interaction, intracellular localization of the CiLV-C proteins with cortical ER/actin network and Golgi, and p29-MP trafficking, we hypothesize the region of replication and transport machinery used for CiLV-C in the cellular infection. We infer that viral replication and particle assembly occur in vesicles (that in several RNA plant viruses represent viral replication complex-VCRs), on cortical ER formed from p24 and p15 ER membrane association. In general, VRCs are formed by RNA, viral proteins and host membrane and proteins, not yet determined for CiLV-C. During viral replication, the p61 (suggested here to be the viral glycoprotein) could be processed in the GC and redirected to VCRs on cortical ER for assembly of the virion envelope. Virus-like particle (VLPs) could be formed by p24 and p61, given their ability in ER membrane remodeling. For trafficking of VRCs and/or ribonucleoprotein complex (vRNP) from the replication site to cell periphery, we suggest different possibilities: (i) the p29, together with p24, would ensure the anchoring of vRNP, VRC or sub-complexes in the actin cytoskeleton, guiding their trafficking along the filaments to cell periphery [1]; (ii) by ER trafficking, where the action of the MP with p29 would facilitate the mobility of the complexes by ER network to neighboring cells, guiding the passage through plasmodesmata [2]; or (iii) by joint action of the p29, MP, and p24 proteins assisting trafficking from both ER/actin network [3]. Whether viral RNA is transferred to neighboring cells through the plasmodesmata by VRC, RNPs or as an entire virus particle from formation of tubular structure, whether viral coat protein participate on viral spread, and whether or not there is a direct involvement of the ER/actin system on viral movement are issues that need to be addressed.

Our findings allow us to hypothesize the possible action of each CiLV-C protein on viral replication, assembly, and movement. Based on our data on cytopathology from infection and protein expression, sublocalization, and membrane association, we propose that viral particle assembly and replication occurs in the endoplasmic reticulum with ER membrane remodeling and vesicle formation from possible action of the p24 and p15 proteins (Figure 10). Similar cellular remodeling with the formation of spherical vesicles has been extensively demonstrated for other viral species, and these structures potentially represent organelle-like compartments for viral replication (Den Boon and Ahlquist, 2010; Verchot, 2011; Ivanov and Mäkinen, 2012). In this sense, p24 and p15 would organize local membrane environment, allowing viral polymerase, in addition to other yet unidentified host and viral proteins, to act for efficient viral replication. In this context, CiLV-C p29 is a strong candidate, given the role of many plant viral coat proteins in viral replication and translation (Ivanov and Mäkinen, 2012), and its plasticity to interact with all other viral proteins assessed (p15, MP, and p24). Although speculative, this observation suggests that some of them, i.e., p24 and p15, may also be potentially involved in this step, given our findings about the functional role of these proteins.

Together with the replication, the p61 protein (glycoprotein) would be processed in the Golgi complex and redirected to VRCs on ER membranes for assembly of the virion envelope (Figure 10). This hypothesis is supported by p61's ability of interacting with GC and to modify the cortical ER; however, further studies are needed to confirm this hypothesis.

Cell-to-cell viral movement encompasses two main categories based on the degree of structural changes they induce in the PD (Scholthof, 2005; Benitez-Alfonso et al., 2010; Niehl and Heinlein, 2011). The first one is that represented by the MP of tobacco mosaic virus (TMV), which interacts with the viral RNA and facilitates the transport of a vRNPs through the PD without causing any visual changes (non-tubule-guided mechanism) (Wolf et al., 1989; Heinlein and Epel, 2004; Niehl and Heinlein, 2011). The other category is represented by the MP of cowpea mosaic virus (CPMV), which forms tubular structures that drastically modify the PD and facilitate the virus passage in the form of virions (tubule-guided mechanism) (Ritzenthaler and Hofmann, 2007). Herein, the results of hetero-association and intracellular localization of the MP, p29, and p24 proteins with cortical ER/actin network, and p29-MP trafficking, provide evidences of how CiLV-C proteins work to ensure its intracellular movement. From these aspects, we suggest different possible manners for CiLV-C intracellular spread: (i) the p29, together with the p24 protein, would ensure the anchoring of vRNP, VRC or sub-complexes in the actin cytoskeleton guiding their trafficking along the filament to cell periphery (Figure 10 [1]); (ii) by ER trafficking, where the action of the MP with p29 would facilitate the mobility of the complexes by ER network to neighboring cells guiding the passage through plasmodesmata (Figure 10 [2]); or (iii) by joint action of the p29, MP, and p24 proteins assisting trafficking from both ER/actin network (Figure 10 [3]). Knowing the degree of participation of the CiLV-C coat protein on the movement would further clarify the mechanism used. In the non-tubule-guided movement, often the movement depends on the MP and may or may not depend on the coat protein (Niehl and Heinlein, 2011). For example, members of the family *Bromoviridae*, the brome mosaic virus (BMV) and cucumber mosaic virus (CMV) move as vRNP and also require CP for viral spread; however, the CP has auxiliary function (Nagano et al., 2001). Carmoviruses do not require CP for their movement, but they depend on two or three specialized MPs (double or triple gene block–TGBp) (Morozov and Solovyev, 2003). Similarly, TMV also does not need its CP for cell-to-cell movement, possibly due to a strong affinity of its MP in binding RNA. On the other hand, viruses that have MPs of low RNA affinity need their CP for movement (Niehl and Heinlein, 2011). Further studies should be performed to evaluate whether or not CiLV-C transport is dependent on nucleocapsid assembly and the direct involvement of the MP with VRC and vRNP transport into the cell.

Since we report the MP co-localization with PD, this protein certainly may assist the viral transport to neighboring cells. In this context, the CiLV-C could be intercellularly transported as vRNP, VRC [VRC may move intracellularly and through PD in association with ER (Kawakami et al., 2004; Hofmann et al., 2009; Sambade and Heinlein, 2009)], as an entire virus particle from formation of tubular structure by polymerization of multiple MP-subunits, or by an intermediated category, were tubular structures promote the movement of ribonucleoprotein complex rather than entire particle (Sánchez-Navarro and Bol, 2001; Sanchez-Navarro et al., 2006; Ritzenthaler and Hofmann, 2007); but this is still an issue that needs to be addressed.

## Author contributions

ML conceived and designed the experiments. ML and EK performed the experiments. ML analyzed and interpreted the data. ML, EK, MS, RR, and JF-A contributed reagents, materials, tools. ML wrote original draft preparation. ML, EK, RR, and JF-A review and editing.

### Conflict of interest statement

The authors declare that the research was conducted in the absence of any commercial or financial relationships that could be construed as a potential conflict of interest.

## Supplementary material

Figure S1 Necrotic reaction from p61 transient expression in *N. benthamiana* leaves. Leaf image of the p29, p15, and p61 CiLV-C proteins transiently expressed in *N. benthamiana* at three days post-infiltration. Necrotic lesion was only visualized by transient expression of the p61:eGFP. As mock controls, leaves were agroinfiltrated with the p29:eGFP and p15:eGFP constructions, where no necrotic lesions were observed in infiltrated tissue. For better visualization of necrosis, leaf expressing the p61:eGFP was treated with a solution of ethanol and acid acetic 3:1 (v/v).

Figure S2 Sub-localization of the proteins containing the N-terminal fused to the eGFP protein. Localization analyses of the p29, p15, p61, MP, and p24 CiLV-C proteins fused at their N-termini with eGFP (•) expressed in epidermal cell of *N. benthamiana*. **(a)** Image of expression of the •-p29 resulting in punctate (white arrow) and inclusion (red arrow) bodies dispersed within the cytoplasm. **(b)** Image of expression of the •-p15 with GFP signal at the nucleus (arrow) and throughout the cytoplasm. **(c)** •-MP expression with fluorescence signal GFP in punctate structures at the cell periphery apparently associated with PD (arrow), and pleomorphic structures in the cytoplasm (red box). **(d)** Image of expression of the •-p24 with fluorescence signal GFP throughout the cytoplasm, apparently associated to the ER (arrow). No spherical structures were visualized; however, filamentous structures are observed (red box). **(e)** Low fluorescence from •-p61 expression. The fluorescence was monitored 72 h post-infiltration (except for p61, 24–36 hpi) using a confocal Leica microscope SP8 model. Bars correspond to 50 μm.

Figure S3 The movement protein (MP) is associated with the plasmodesmata. Expression of MP carrying the GFP (•) in *N. benthamiana* leaves at 72 h post infiltration. The callose deposits were stained using aniline blue (•). The MP punctate structures co-localize at the cell periphery with the fluorochrome, suggesting accumulation of the MP at the plasmodesmata. From the left to the right, the green (GFP), blue (Aniline blue), transmitted light channels, and the merged image are shown in the figure. In a higher magnification image it is shown the co-localization of the MP with callose deposits in the plasmodesmata, and the chart of fluorescence intensities further confirms the co-localization. Distance measurement starts from the base to the tip of the arrows (x axis). The mean SD of Person Correlation Coefficient (PCC) is given in the merged image. The dotted line in the transmitted light image indicates the cell wall (CW). Red and blue bars correspond to 10 and 5 μm, respectively.

Figure S4 Hydrophobic prediction analyses of the CiLV-C proteins. **(A)** Transmembrane region and Coiled coil analyses of the p29, p15, p61, MP, and p24 proteins using different computational tools. For p29, p15, and MP TMDs were not identified, whereas for p61 it was predicted one or three or five TMDs, and for p24, four TMDs. Coiled coil structures were only predicted for p29 and p24. **(B)** Hydrophobic regions (HR) were predicted for p29, p15, MP, and p24. A schematic representation of the proteins highlighting the HRs can be found at the top of each picture (in orange). Hydrophobic profile of the proteins is shown in the graphics generated with MPEx tool. The red lines show the mean values using a window of 19 residues and the yellow line indicates the predicted HRs. Values of ΔG of the TMD are indicated for p24. **(C)** Coiled coil domains of the p29 and p24 proteins were identified by PCOILS server. The green, blue and purple lines represent the results obtained with a window 14, 21, and 28, respectively.

Table S1 Nuclear localization/exportation signals, signal peptide, and N-glycosylation analyses of the CiLV-C proteins using computational tools.

Movie S1 Citrus leprosis virus C p29:eGFP punctate and inclusion bodies trafficking throughout the cytoplasm in *N. benthamiana* cells. Arrows mark the position of the p29 structure at each time point. Punctate sturtures shows a fast mobility (red arrows), while inclusion shows a slower movement (white arrows; (avi).

Movie S2 Intracellular mobility of the CiLV-C MP:eGFP filamentous aggregates in *N. benthamiana* cells (avi).

Movie S3 Citrus leprosis virus C p29:eGFP structures trafficking along the cortical ER in *N. benthamiana* cells. Arrows mark the position of the p29 structure at each time point trafficking along the cortical ER. Blue, green and yellow arrows mark individuals p29:eGFP punctate bodies, which merge into a larger aggregate (inclusions) represented by red arrow (avi).

Movie S4 Citrus leprosis virus C MP:eGFP filamentous aggregates trafficking along the cortical ER in *N. benthamiana* cells. The white arrows highlight a region of continuous movement of the MP structures (avi).
