# Supplementary figures and images for: Dissecting the Subcellular Localization, Intracellular Trafficking, Interactions, Membrane Association, and Topology of Citrus Leprosis Virus C Proteins
Source: Front Plant Sci. 2018 Sep 11;9:1299. doi: 10.3389/fpls.2018.01299 (PMC6141925; doi:10.3389/fpls.2018.01299)

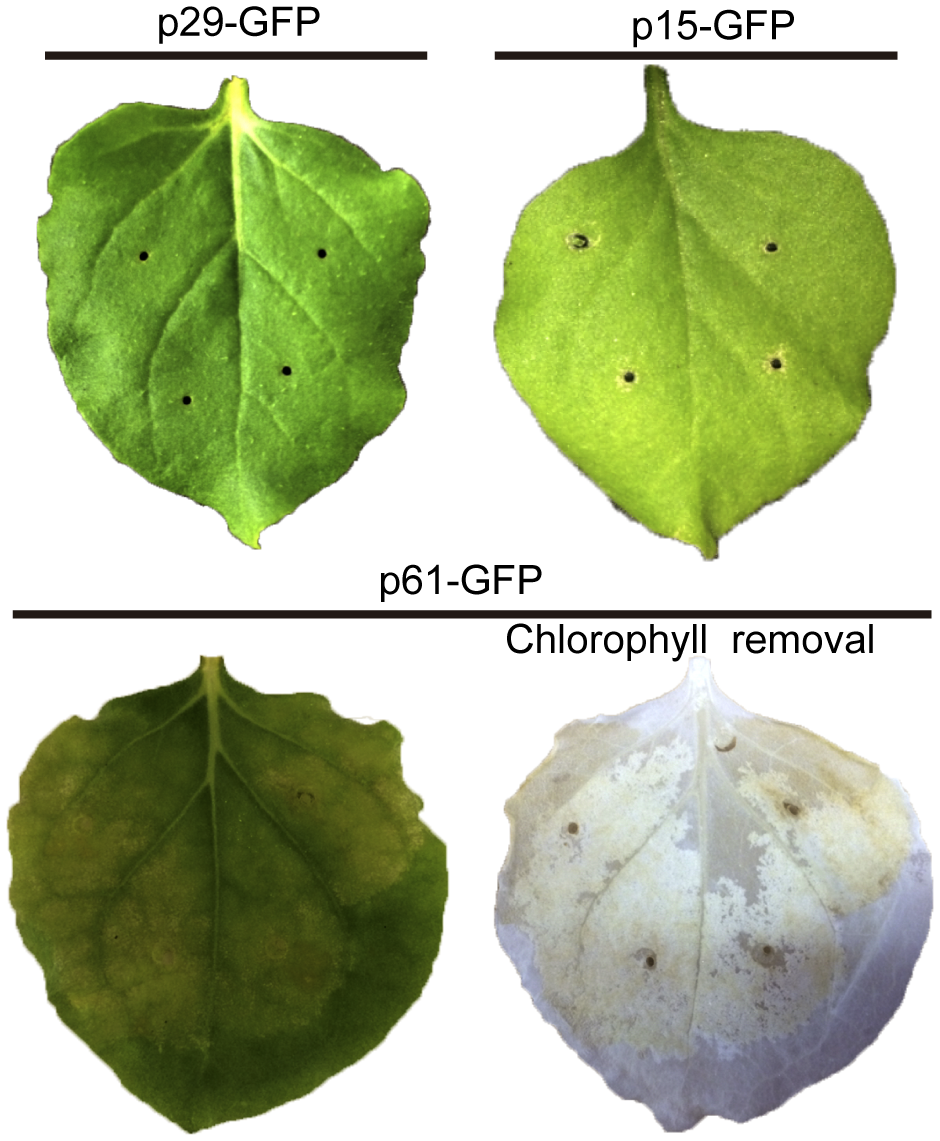

Supplement: Figure S1 — Necrotic reaction from p61 transient expression in N. benthamiana leaves. Leaf image of the p29, p15, and p61 CiLV-C proteins transiently expressed in N. benthamiana at three days post-infiltration. Necrotic lesion was only visualized by transient expression of the p61:eGFP. As mock controls, leaves were agroinfiltrated with the p29:eGFP and p15:eGFP constructions, where no necrotic lesions were observed in infiltrated tissue. For better visualization of necrosis, leaf expressing the p61:eGFP was treated with a solution of ethanol and acid acetic 3:1 (v/v). [file Image_1.TIF]

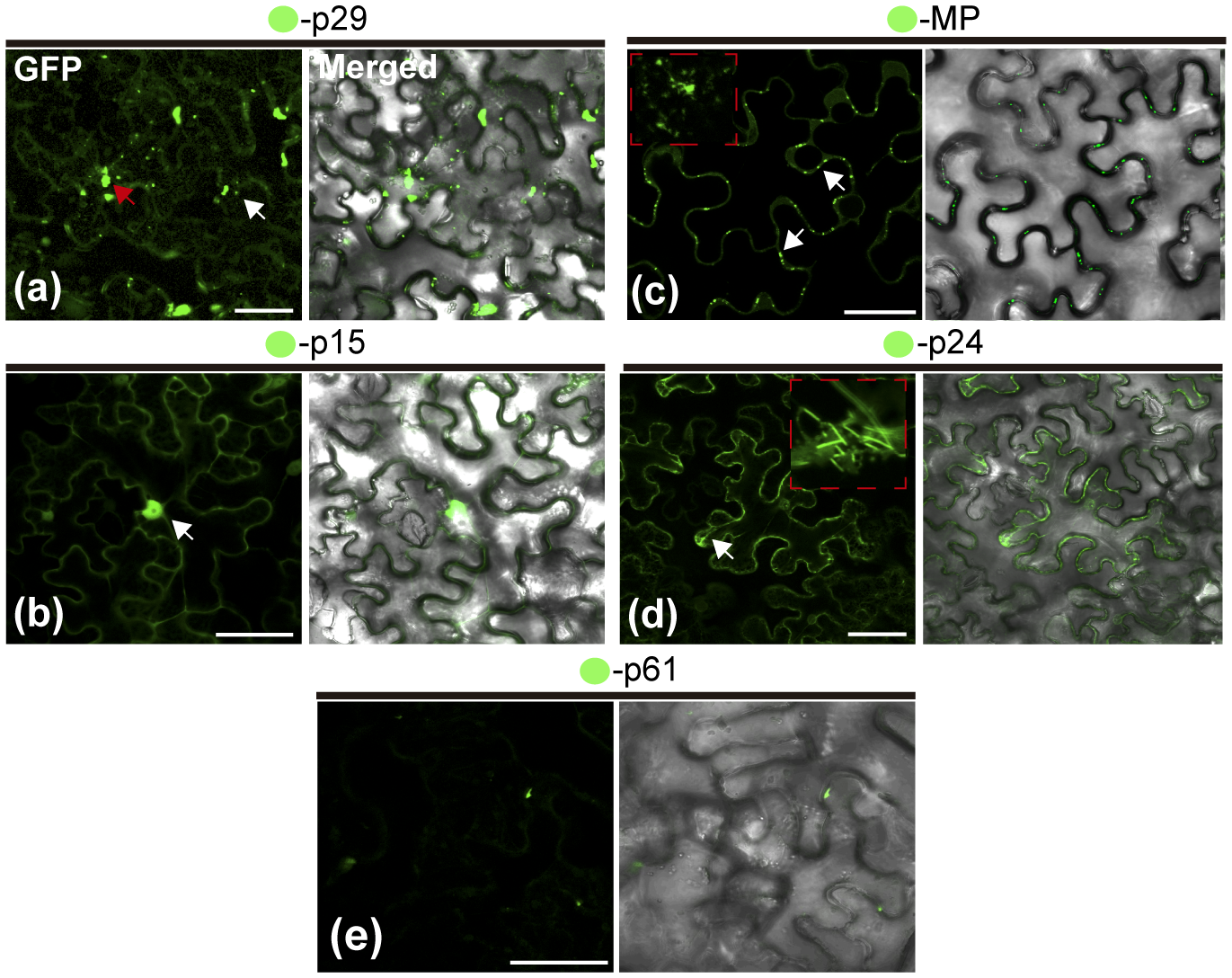

Supplement: Figure S2 — Sub-localization of the proteins containing the N-terminal fused to the eGFP protein. Localization analyses of the p29, p15, p61, MP, and p24 CiLV-C proteins fused at their N-termini with eGFP (•) expressed in epidermal cell of N. benthamiana. (a) Image of expression of the •-p29 resulting in punctate (white arrow) and inclusion (red arrow) bodies dispersed within the cytoplasm. (b) Image of expression of the •-p15 with GFP signal at the nucleus (arrow) and throughout the cytoplasm. (c) •-MP expression with fluorescence signal GFP in punctate structures at the cell periphery apparently associated with PD (arrow), and pleomorphic structures in the cytoplasm (red box). (d) Image of expression of the •-p24 with fluorescence signal GFP throughout the cytoplasm, apparently associated to the ER (arrow). No spherical structures were visualized; however, filamentous structures are observed (red box). (e) Low fluorescence from •-p61 expression. The fluorescence was monitored 72 h post-infiltration (except for p61, 24–36 hpi) using a confocal Leica microscope SP8 model. Bars correspond to 50 μm. [file Image_2.TIF]

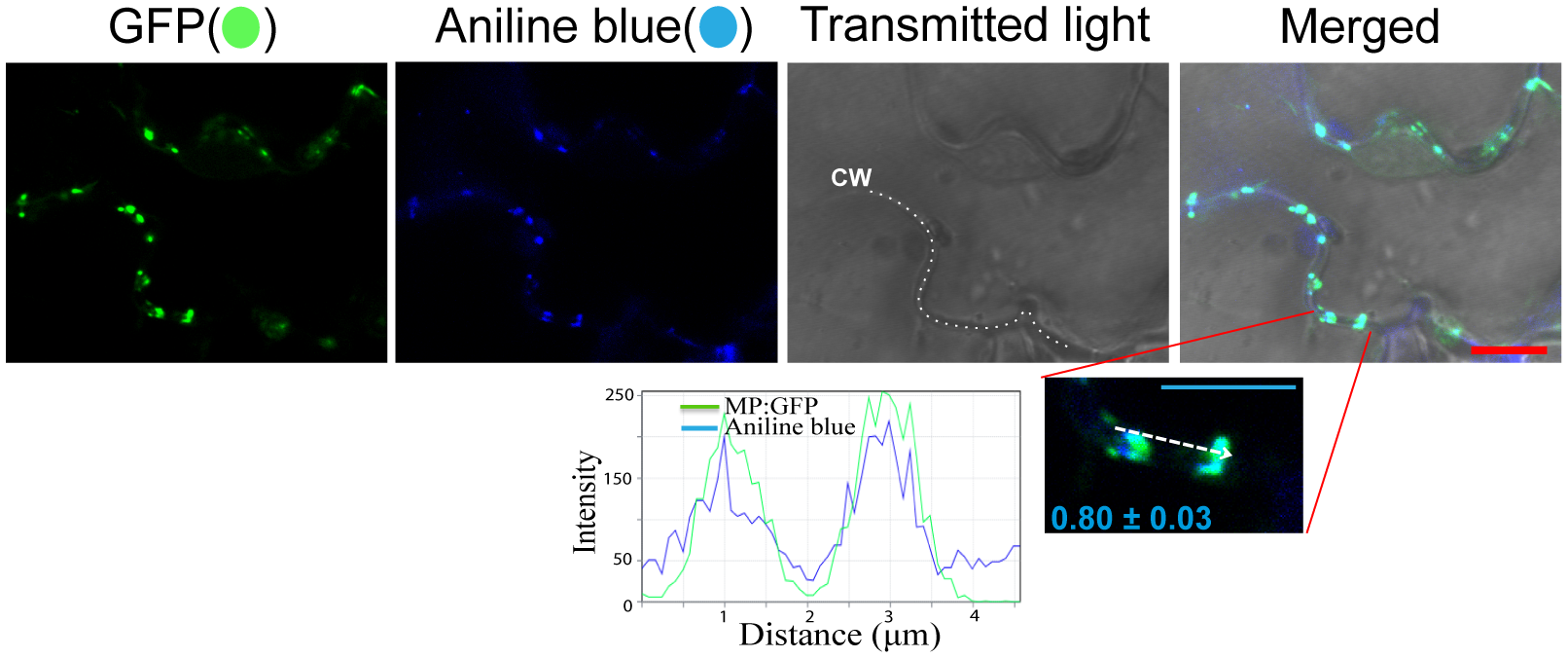

Supplement: Figure S3 — The movement protein (MP) is associated with the plasmodesmata. Expression of MP carrying the GFP (•) in N. benthamiana leaves at 72 h post infiltration. The callose deposits were stained using aniline blue (•). The MP punctate structures co-localize at the cell periphery with the fluorochrome, suggesting accumulation of the MP at the plasmodesmata. From the left to the right, the green (GFP), blue (Aniline blue), transmitted light channels, and the merged image are shown in the figure. In a higher magnification image it is shown the co-localization of the MP with callose deposits in the plasmodesmata, and the chart of fluorescence intensities further confirms the co-localization. Distance measurement starts from the base to the tip of the arrows (x axis). The mean SD of Person Correlation Coefficient (PCC) is given in the merged image. The dotted line in the transmitted light image indicates the cell wall (CW). Red and blue bars correspond to 10 and 5 μm, respectively. [file Image_3.TIF]

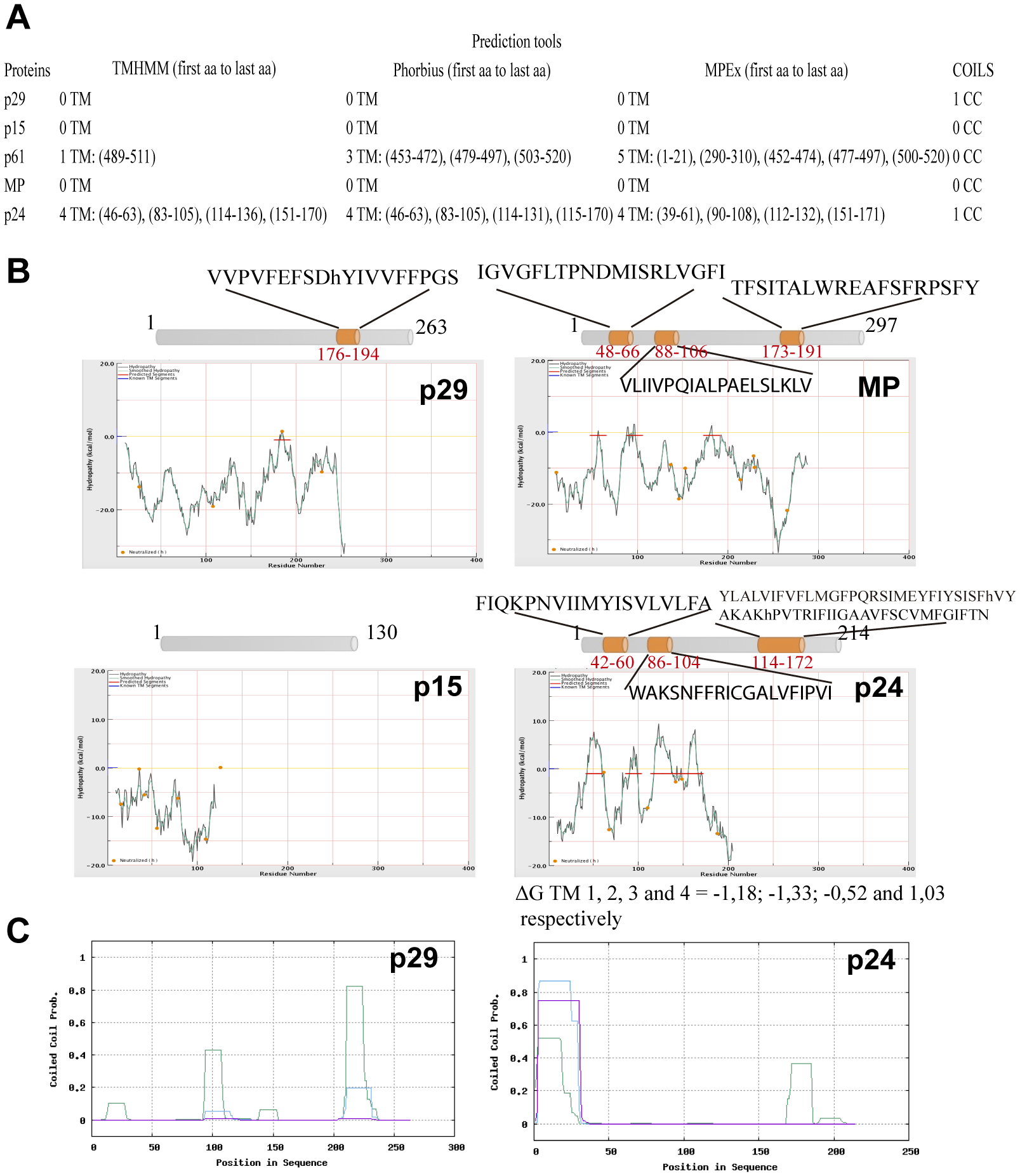

Supplement: Figure S4 — Hydrophobic prediction analyses of the CiLV-C proteins. (A) Transmembrane region and Coiled coil analyses of the p29, p15, p61, MP, and p24 proteins using different computational tools. For p29, p15, and MP TMDs were not identified, whereas for p61 it was predicted one or three or five TMDs, and for p24, four TMDs. Coiled coil structures were only predicted for p29 and p24. (B) Hydrophobic regions (HR) were predicted for p29, p15, MP, and p24. A schematic representation of the proteins highlighting the HRs can be found at the top of each picture (in orange). Hydrophobic profile of the proteins is shown in the graphics generated with MPEx tool. The red lines show the mean values using a window of 19 residues and the yellow line indicates the predicted HRs. Values of ΔG of the TMD are indicated for p24. (C) Coiled coil domains of the p29 and p24 proteins were identified by PCOILS server. The green, blue and purple lines represent the results obtained with a window 14, 21, and 28, respectively. [file Image_4.TIF]
